# Presynaptic structural and functional plasticity are coupled by convergent Rap1 signaling

**DOI:** 10.1083/jcb.202309095

**Published:** 2024-05-15

**Authors:** Yeongjin David Kim, Hyun Gwan Park, Seunghwan Song, Joohyung Kim, Byoung Ju Lee, Kendal Broadie, Seungbok Lee

**Affiliations:** 1 Department of Brain and Cognitive Sciences, Seoul National University, Seoul, Korea; 2 Interdisciplinary Program in Neuroscience, Seoul National University, Seoul, Korea; 3 Department of Cell and Developmental Biology and Dental Research Institute, Seoul National University, Seoul, Korea; 4 Departments of Cell and Developmental Biology, Pharmacology, and Biological Sciences, Vanderbilt University and Medical Center, Nashville, TN, USA

## Abstract

Dynamic presynaptic actin remodeling drives structural and functional plasticity at synapses, but the underlying mechanisms remain largely unknown. Previous work has shown that actin regulation via Rac1 guanine exchange factor (GEF) Vav signaling restrains synaptic growth via bone morphogenetic protein (BMP)-induced receptor macropinocytosis and mediates synaptic potentiation via mobilization of reserve pool vesicles in presynaptic boutons. Here, we find that Gef26/PDZ-GEF and small GTPase Rap1 signaling couples the BMP-induced activation of Abelson kinase to this Vav-mediated macropinocytosis. Moreover, we find that adenylate cyclase Rutabaga (Rut) signaling via exchange protein activated by cAMP (Epac) drives the mobilization of reserve pool vesicles during post-tetanic potentiation (PTP). We discover that Rap1 couples activation of Rut-cAMP-Epac signaling to Vav-mediated synaptic potentiation. These findings indicate that Rap1 acts as an essential, convergent node for Abelson kinase and cAMP signaling to mediate BMP-induced structural plasticity and activity-induced functional plasticity via Vav-dependent regulation of the presynaptic actin cytoskeleton.

## Teaser

Dynamic remodeling of actin cytoskeletal networks is critical for many aspects of presynaptic plasticity. However, it remains poorly defined how presynaptic actin is regulated. Kim et al. demonstrate that the presynaptic Abl-Gef26 and Rut-cAMP-Epac pathways converge on actin-regulatory Rap1-Vav-Rac1 signaling to mediate Gbb/BMP-induced presynaptic growth and activity-induced functional plasticity, respectively.

## Introduction

Synaptic plasticity is driven by the dynamic remodeling of the actin cytoskeleton (Cingolani and Goda, 2008; Gentile et al., 2022; Rust and Maritzen, 2015). The *Drosophila* neuromuscular junction (NMJ) provides a powerful model system for studying the molecular basis of this plasticity (Bai and Suzuki, 2020; Menon et al., 2013). These synapses manifest experience- and activity-dependent plasticity while also continuously expanding during larval development to scale with the enormous muscle growth (Schuster et al., 1996). Synaptic scaling during development critically depends on the retrograde signal Glass bottom boat (Gbb), a muscle-derived ligand of the bone morphogenetic protein (BMP) family (McCabe et al., 2003). The Gbb ligand binds and activates BMP receptors (BMPRs) in the presynaptic motor neuron terminal (Aberle et al., 2002; Marques et al., 2002; Rawson et al., 2003). This signaling, in turn, leads to the activation of the Mothers against decapentaplegic (Mad) transcription factor to regulate target genes controlling synaptic architecture. Our previous results show that Gbb activates Abelson (Abl) tyrosine kinase, inducing macropinocytosis and subsequent intracellular degradation of presynaptic BMPRs, which generates a negative feedback mechanism to limit Gbb-dependent synaptic growth (Kim et al., 2019). This homeostatic process is dependent on presynaptic actin cytoskeleton remodeling mediated by the Rac1-specific guanine exchange factor (GEF) Vav (Kim et al., 2019; Park et al., 2022). However, the signaling mechanisms by which Abl kinase activates Vav-Rac1 signaling during presynaptic macropinocytosis have not previously been identified.

In addition to structural plasticity, the *Drosophila* NMJ exhibits activity-dependent functional plasticity, including post-tetanic potentiation (PTP), a widespread but rather poorly understood form of short-term potentiation resulting from the enhancement of neurotransmitter quantal release from synaptic vesicles (SVs) (Broadie et al., 1997; Zhong and Wu, 1991). PTP is primarily triggered by presynaptic Ca^2+^ accumulation during repetitive firing depolarization (Regehr, 2012). PTP is strongly impaired by loss-of-function mutations in the *rutabaga* (*rut*) gene encoding a Ca^2+^/calmodulin-regulated adenylate cyclase that synthesizes cyclic AMP (cAMP; Zhong and Wu, 1991). PTP is also known to require the recruitment of SVs from the reserve pool (RP) to the exo-endo cycling pool (ECP; Kim et al., 2009), with tetanic stimulation triggering cAMP-mediated RP mobilization (Kuromi and Kidokoro, 2000). Elevation of presynaptic cAMP levels by forskolin (FSK), an adenylyl cyclase activator, is sufficient to enhance baseline synaptic transmission at the *Drosophila* NMJ and mammalian synapses (Cheung et al., 2006; Fernandes et al., 2015; Gekel and Neher, 2008; Kaneko and Takahashi, 2004). Although these findings demonstrate the importance of cAMP signaling to functional synaptic plasticity, its role in the production of PTP has not been directly addressed. Importantly, presynaptic Vav-Rac1 signaling has been shown to mediate PTP by driving the mobilization of RP vesicles (Park et al., 2022), suggesting that Rut-dependent cAMP signaling may act through Vav/Rac1 regulation of presynaptic actin dynamics.

FSK/cAMP-induced synaptic potentiation at both invertebrate and vertebrate synapses is frequently mediated by exchange protein activated by cAMP (Epac; Fernandes et al., 2015; Gekel and Neher, 2008; Kaneko and Takahashi, 2004; Zhong and Zucker, 2005), an activator of the small GTPase Rap1 (de Rooij et al., 1998; Enserink et al., 2002; Kawasaki et al., 1998). In *Drosophila*, Rap1 also serves as a target of the PDZ-GEF homolog Gef26 (Huelsmann et al., 2006; Spahn et al., 2012; Wang et al., 2006). Presynaptic Gef26 and Rap1 together act to restrain synaptic growth at the *Drosophila* NMJ by attenuating retrograde Gbb signaling (Heo et al., 2017), further supporting a functional link between these two pathways. In this study, we tested the differential functions of the Gef26- and Epac-mediated Rap1 pathways at the *Drosophila* NMJ. We found that Abl kinase activation mimics Gbb-induced presynaptic macropinocytosis requiring both Gef26 and Rap1 to regulate synaptic growth. Rap1, but not Abl or Gef26, also mediates cAMP-dependent RP vesicle mobilization and PTP, with FSK and tetanic stimulation similarly increasing presynaptic vesicle trafficking. Rut and Epac are likewise required, with the synaptic potentiation induced by FSK and mediated by Rap1 dependent on the presynaptic F-actin cytoskeleton. Our results suggest that the Gef26-Rap1 pathway couples Gbb-induced Abl signaling to Vav-mediated presynaptic macropinocytosis to restrain synaptic growth, whereas the Epac-Rap1 pathway couples Rut-dependent cAMP signaling to Vav-mediated induction of RP vesicle mobilization to mediate synaptic potentiation.

## Results

### Activation of Abl kinase induces presynaptic macropinocytosis to mimic BMP signaling

**Figure 1.**
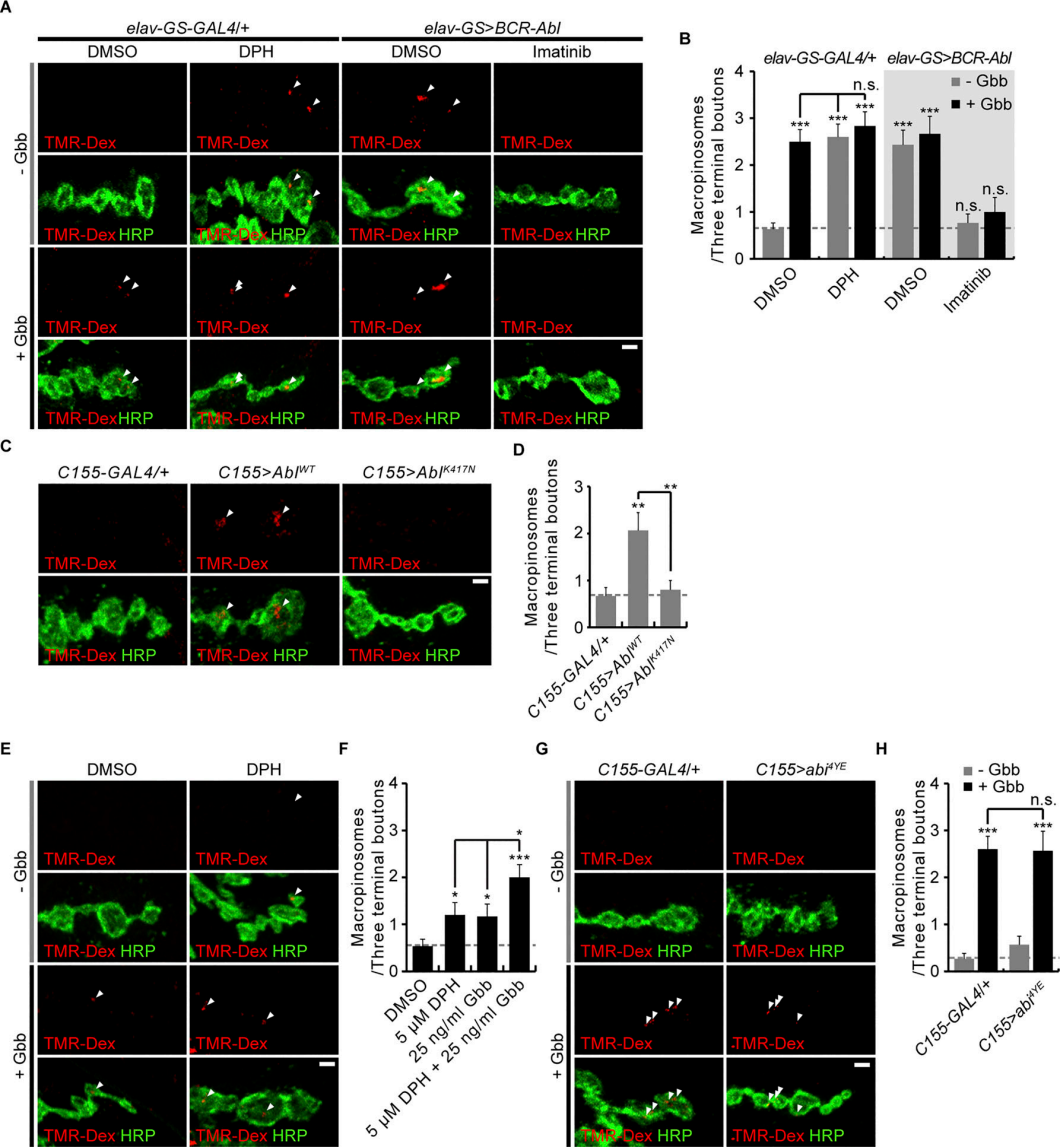
**Abelson kinase activation, but not Abl-mediated phosphorylation of Abi, mimics Gbb-induced presynaptic macropinocytosis. (A)** Representative images of NMJ 6/7 terminals of RU486-fed *elav-GS-GAL4/+* and *UAS-BCR-Abl/+*; *elav-GS-GAL4/+* (*elav-GS*>*BCR-Abl*) third instar larvae labeled with anti-HRP (green) following a 5-min pulse of TMR-Dex (red, 2 mg/ml) in the absence/presence of Gbb (50 ng/ml). Indicated preparations were pretreated with DPH (10 μM) or imatinib (50 μM) for 30 min. **(B)** Quantification of the number of TMR-Dex-positive puncta per three terminal boutons. **(C)** Representative NMJ 6/7 images of *C155-GAL4/+*, *C155-GAL4/+*; *UAS-Abl*^*WT*^/+ (*C155*>*Abl*^*WT*^), and *C155-GAL4/+*; *UAS-Abl*^*K417N*^/+ (*C155*>*Abl*^*K417N*^) labeled with anti-HRP (green) following a 5-min pulse of TMR-Dex (red, 2 mg/ml) in the absence of Gbb. **(D)** Quantification of the number of TMR-Dex-positive puncta per three terminal boutons. **(E)** Representative NMJ 6/7 images of WT labeled with anti-HRP (green) following a 5-min pulse of TMR-Dex (red, 2 mg/ml) in the absence/presence of Gbb (25 ng/ml) and DPH (5 μM). **(F)** Quantification of the number of TMR-Dex-positive puncta per three terminal boutons. **(G)** Representative NMJ 6/7 images of *C155-GAL4/+* and *C155-GAL4/+*; *UAS-HA-abi*^*4YE*^*/+* (*C155*>*abi*^*4YE*^) labeled with anti-HRP (green) following a 5-min pulse of TMR-Dex (red, 2 mg/ml) in the absence/presence of Gbb (50 ng/ml). **(H)** Quantification of the number of TMR-Dex-positive puncta per three terminal boutons. *n* = 30 NMJ branches. Data represent mean ± SEM. Comparisons are with unstimulated WT (DMSO), *elav-GS-GAL4*/+, or *C155-GAL4*/+ control (*, P < 0.05; **, P < 0.01; ***, P < 0.001; n.s., not significant by one-way ANOVA with multiple comparisons). Arrowheads indicate TMR-Dex-positive puncta within HRP-labeled synaptic boutons. Scale bars: 2 μm.

At the *Drosophila* NMJ, the BMP signaling ligand Gbb potently induces presynaptic macropinocytosis via activation of Abelson (Abl) kinase and the Vav-Rac1-SCAR/WAVE pathway (Kim et al., 2019; Park et al., 2022). To further define the role of Abl in this mechanism, we tested whether Abl kinase activation is sufficient to induce presynaptic macropinocytosis using a classic macropinocytic tracer, 70-kDa dextran conjugated to tetramethylrhodamine (TMR-Dex; Kim et al., 2019). Application of a saturating concentration of Gbb (50 ng/ml) causes a 395 ± 41% increase in TMR-Dex internalization into the presynaptic terminal of the third instar larval NMJ (Fig. 1, A and B). This effect was fully mimicked by either application of a saturating concentration of the cell-permeable Abl activator 5-(1,3-diaryl-1H-pyrazol-4-yl)hydantoin (DPH; 10 μM) or neuronal *elav-GS-GAL4*-driven overexpression of human/*Drosophila* chimeric P210 BCR-Abl (BCR-Abl; Fig. 1, A and B), which possesses constitutive kinase activity (Fogerty et al., 1999). The stimulatory effect of BCR-Abl overexpression on TMR-Dex internalization is completely abrogated by the tyrosine kinase inhibitor imatinib (50 μM; Fig. 1, A and B). These findings indicate that activation of Abl kinase activity is sufficient to trigger presynaptic macropinocytosis, even in the absence of Gbb stimulation. This conclusion is confirmed by comparing the effects of neuronal *C155-GAL4*-driven overexpression of *Drosophila* Abl and a kinase-dead mutant (Henkemeyer et al., 1990). Indeed, overexpression of WT Abl is able to potently induce TMR-Dex internalization while overexpression of Abl^K417N^ has no effect (Fig. 1, C and D). Taken together, these results indicate that Gbb-induced macropinocytosis is due to the activation of Abl kinase.

To further corroborate this conclusion, we also tested whether Abl and Gbb signaling interact with one another to induce presynaptic macropinocytosis. Application of a lower concentration of either DPH (5 μM) or Gbb (25 ng/ml) causes a half-maximal induction of TMR-Dex internalization (DPH, 225 ± 50%; Gbb, 219 ± 50%; Fig. 1, E and F). Notably, the coapplication of 5 μM DPH and 25 ng/ml Gbb causes a submaximal increase in TMR-Dex internalization (375 ± 51%; Fig. 1, E and F). By contrast, this additive effect is completely lost when saturating concentrations of DPH (10 μM) and Gbb (50 ng/ml) are coapplied (Fig. 1, A and B). Similarly, BCR-Abl overexpression abrogates the stimulatory effect of 50 ng/ml Gbb on TMR-Dex internalization (Fig. 1, A and B). These results strongly support the conclusion that Gbb-induced presynaptic macropinocytosis is due to the activation of Abl kinase activity.

Previous work has shown that Abl-mediated phosphorylation of Abelson interactor (Abi), an integral component of the SCAR complex, is necessary for Gbb-induced, Vav-Rac1-SCAR-mediated presynaptic macropinocytosis (Kim et al., 2019). To test whether this posttranslational modification is also sufficient to induce presynaptic macropinocytosis, we overexpressed Abi^4YE^, a phosphomimic variant of Abi with mutated sites of Abl phosphorylation (148, 155, 248, 285; Huang et al., 2007; Kim et al., 2019). Neuronal overexpression of *UAS-abi*^*4YE*^ using *C155-GAL4* fails to induce any TMR-Dex internalization into presynaptic NMJ terminals in the absence of Gbb or to block the stimulatory effect of Gbb signaling (Fig. 1, G and H). Combined with the effects of DPH and BCR-Abl, these results indicate that, in addition to Abi phosphorylation, Abl may employ additional mechanisms to activate Vav-Rac1-SCAR signaling during Gbb-induced presynaptic macropinocytosis.

Consistent with the role of presynaptic macropinocytosis in attenuating retrograde Gbb signaling during synaptic growth, loss-of-function mutations in *Abl*, *Vav*, and *Rac1* induce NMJ structural overgrowth with an excess number of satellite boutons (Kim et al., 2019; Park et al., 2022), a phenotypic hallmark of excessive presynaptic Gbb signaling (Nahm et al., 2013; O’Connor-Giles et al., 2008). A similar phenotype was also induced by the loss of Gef26/PDZ-GEF, or its downstream effector Rap1 (Heo et al., 2017). In mammalian cells, Rap1 promotes Rac1-mediated cell spreading by localizing Vav2 to sites of lamellipodia extension (Arthur et al., 2004). These findings suggest that the Gef26-Rap1 pathway might provide a functional link between Abl and Vav during Gbb-induced macropinocytosis and synaptic growth at the NMJ. We next tested this hypothesis using several approaches.

### The Gef26-Rap1 pathway links Abl and Vav signaling to presynaptic growth

**Figure 2.**
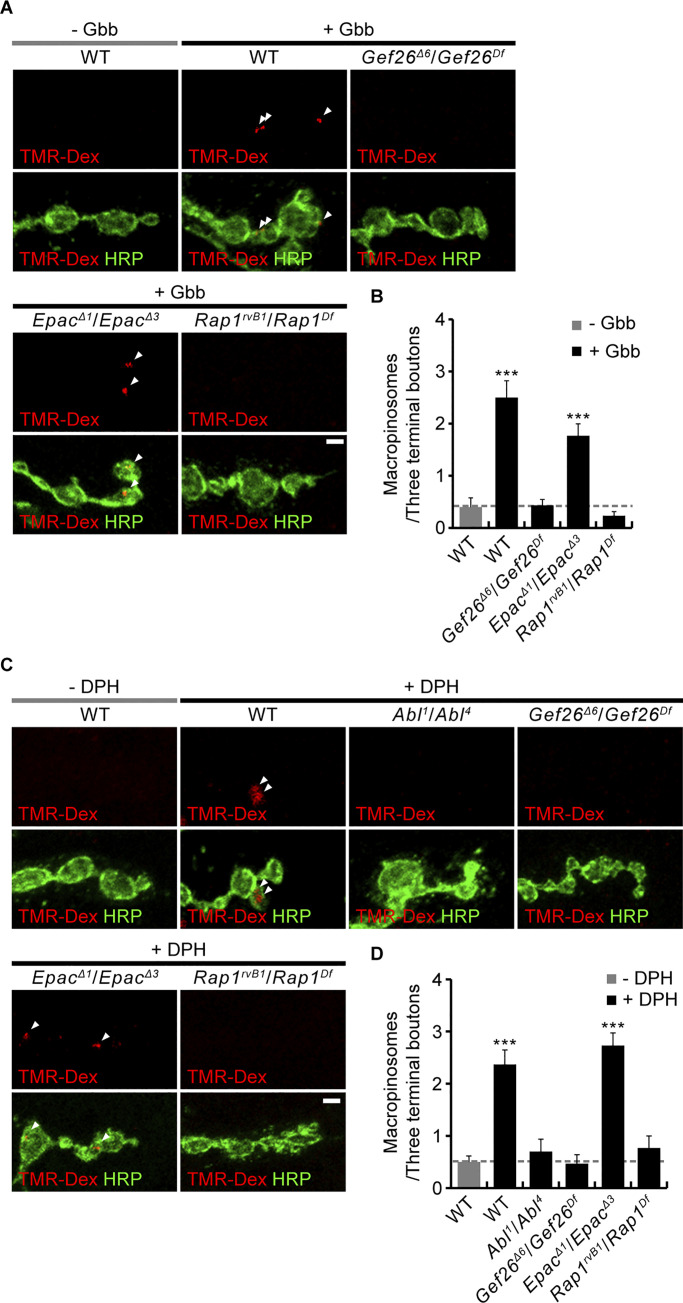
**Rap1 and Gef26 are required for Gbb-induced, Abl-mediated macropinocytosis. (A)** Representative NMJ 6/7 images of WT, *Gef26*^*Δ6*^*/Gef26*^*Df*^, *Epac*^*Δ1*^*/Epac*^*Δ3*^, and *Rap1*^*rvB1*^*/Rap1*^*Df*^ larvae labeled with anti-HRP (green) following a 5-min pulse of TMR-Dex (red, 2 mg/ml) in the absence/presence of Gbb (50 ng/ml). **(B)** Quantification of the number of TMR-Dex puncta per three terminal boutons. **(C)** Representative NMJ 6/7 images of WT, *Abl*^*1/4*^, *Gef26*^*Δ6*^*/Gef26*^*Df*^, *Epac*^*Δ1*^*/Epac*^*Δ3*^, and *Rap1*^*rvB1*^*/Rap1*^*Df*^ larvae labeled with anti-HRP (green) following a 5-min pulse of TMR-Dex (red, 2 mg/ml) with/without DPH pretreatment (10 μM). Arrowheads indicate TMR-Dex puncta within HRP-labeled presynaptic boutons. **(D)** Quantification of the number of TMR-Dex puncta per three terminal boutons. *n* = 30 NMJ branches. Data represent mean ± SEM. Comparisons are with unstimulated WT control (***, P < 0.001 by one-way ANOVA with multiple comparisons). Arrowheads indicate TMR-Dex puncta in presynaptic boutons. Scale bars: 2 μm.

**Figure 3.**
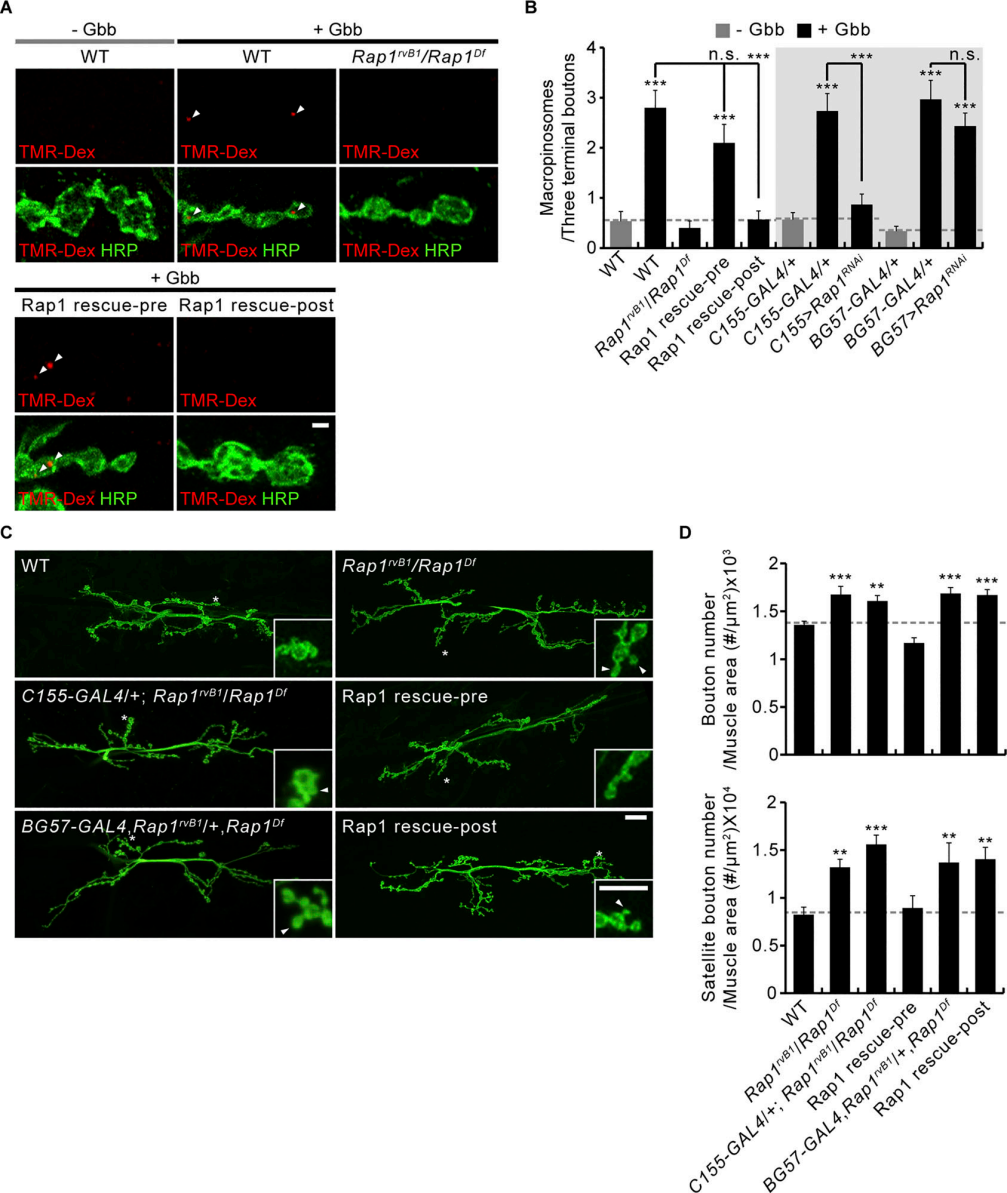
**Presynaptic Rap1 controls macropinocytosis and synaptic growth. (A)** Representative NMJ 6/7 images of WT, *Rap1*^*rvB1*^/*Rap1*^*Df*^, *C155-GAL4*/+; *Rap1*^*rvB1*^,+/*UAS*-*Rap1*,*Rap1*^*Df*^ (Rap1 rescue-pre), *BG57*-*GAL4*,*Rap1*^*rvB1*^/*UAS*-*Rap1*,*Rap1*^*Df*^ (Rap1 rescue-post), *C155-GAL4*/+, *C155-GAL4*/+; *UAS-Rap1*^*RNAi*^/+ (*C155*>*Rap1*^*RNAi*^), *BG57-GAL4*/+, and *BG57-GAL4*/+; *UAS-Rap1*^*RNAi*^/+ (*BG57*>*Rap1*^*RNAi*^) labeled with anti-HRP (green) following a 5 min pulse of TMR-Dex (red, 2 mg/ml) in the absence/presence of Gbb (50 ng/ml). Arrowheads indicate TMR-Dex-positive puncta in HRP-labeled presynaptic terminals. **(B)** Quantification of the number of TMR-Dex-positive puncta per three terminal boutons (*n* = 30 NMJ branches). **(C)** Representative NMJ 6/7 images of WT, *Rap1*^*rvB1*^/*Rap1*^*Df*^, *C155-GAL4*/+; *Rap1*^*rvB1*^/*Rap1*^*Df*^, *C155-GAL4*/+; *Rap1*^*rvB1*^,+/*Rap1*^*Df*^,*UAS*-*Rap1* (Rap1 rescue-pre), *BG57*-*GAL4*,*Rap1*^*rvB1*^/+,*Rap1*^*Df*^, and *BG57*-*GAL4*,*Rap1*^*rvB1*^/*UAS*-*Rap1*,*Rap1*^*Df*^ (Rap1 rescue-post) labeled with anti-HRP. Insets show higher magnification views of the areas marked by asterisks. Satellite boutons budding off axon terminal arbors are indicated by arrowheads. **(D)** Quantification of total and satellite bouton numbers normalized to muscle surface area at NMJ 6/7 (*n* = 18 NMJs). Data represent mean ± SEM. Comparisons are with the wild type unless otherwise indicated (**, P < 0.01; ***, P < 0.001; n.s., not significant by one-way ANOVA with multiple comparisons). Scale bars: 2 μm (A); 20 μm (C); 10 μm (C, inset).

**Figure S1.**
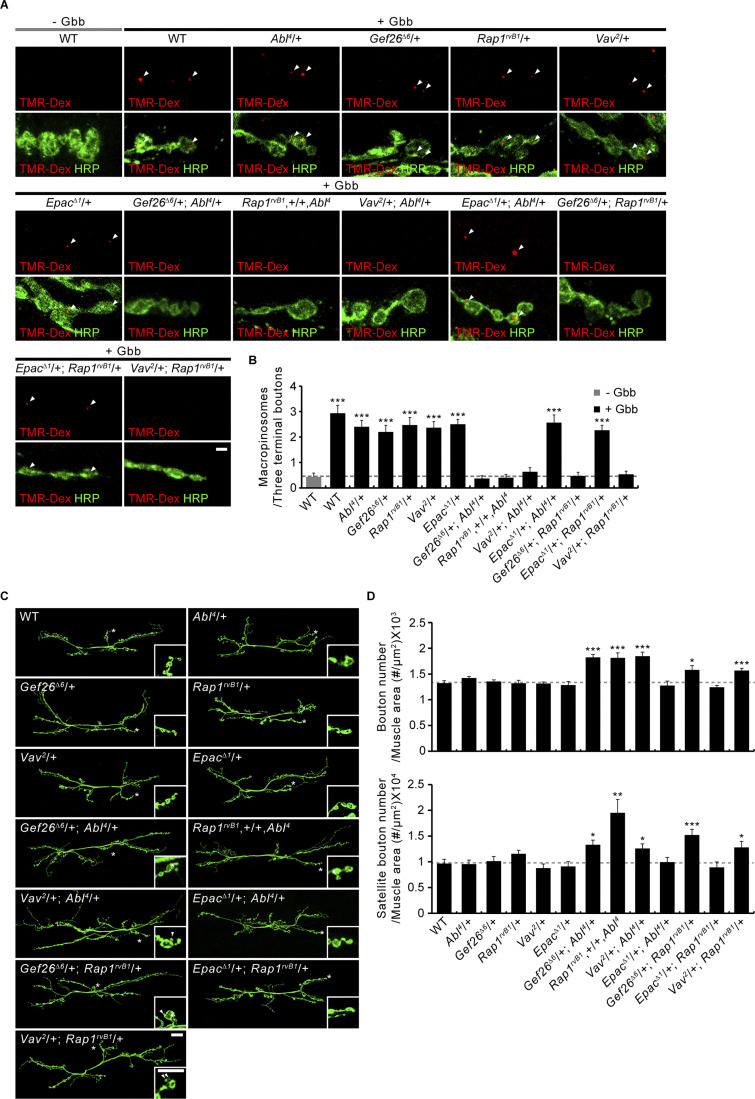
**Transheterozygous interactions among *Abl*, *Gef26*, *Epac*, *Rap1*, and *Vav* during Gbb-induced presynaptic macropinocytosis and synaptic growth. (A)** Representative NMJ 6/7 images of third instar larvae of indicated genotypes labeled with anti-HRP (green) following a 5-min pulse with TMR-Dex (red, 2 mg/ml) containing Gbb (50 ng/ml). Arrowheads indicate TMR-Dex-positive puncta within HRP-labeled presynaptic terminals. Scale bar, 2 μm. **(B)** Quantification of the number of TMR-Dex-positive puncta per bouton (*n* = 30 NMJ branches). Data represent mean ± SEM. Comparisons are with Gbb-unstimulated wild type (***, P < 0.001 by one-way ANOVA with multiple comparisons). **(C)** Representative NMJ 6/7 images of third instar larvae of indicated genotypes labeled with anti-HRP. Insets show higher magnification views of the areas marked by asterisks. Arrowheads indicate satellite boutons budding off axon terminal arbors. Scale bar: 20 μm. **(D)** Quantification of total and satellite bouton numbers normalized to muscle area. *n* = 15 NMJs. Data represent mean ± SEM. Comparisons are with the wild type (*, P < 0.05; **, P < 0.01; ***, P < 0.001 by one-way ANOVA with multiple comparisons).

We first tested the effect of depleting Gef26 or Rap1 on both Gbb- and DPH-induced macropinocytosis at the *Drosophila* NMJ. We find that loss of Gef26 or Rap1 completely abrogates Gbb- and DPH-induced TMR-Dex uptake by presynaptic NMJ terminals (Fig. 2). By contrast, loss of another Rap1-GEF Epac has no effect on Gbb- and DPH-induced TMR-Dex uptake (Fig. 2), demonstrating a specific role of Gef26-dependent Rap1 signaling in Gbb-induced, Abl-mediated presynaptic macropinocytosis. Expression of *UAS-Rap1* in *Rap1* mutants restores Gbb-induced macropinocytosis to WT levels (Fig. 3, A and B). In contrast, muscle expression of *UAS-Rap1* using *BG57-GAL4* does not rescue the defect in Gbb-induced macropinocytosis. This phenotype of *Rap1* mutants is recapitulated by presynaptic, but not postsynaptic, expression of *UAS-Rap1*^*RNAi*^ in the WT background (Fig. 3 B), confirming the presynaptic requirement for Rap1 in Gbb-induced presynaptic macropinocytosis. We next tested whether there are transheterozygous interactions among *Abl*, *Gef26*, *Epac*, *Rap1*, and *Vav* during Gbb-induced macropinocytosis to define the signaling pathway. Although Gbb-induced presynaptic macropinocytosis normally occurs in animals heterozygous for a null allele of *Abl*, *Gef26*, *Rap1*, or *Vav*, it is completely impaired in animals transheterozygous for *Abl* and *Gef26*, *Rap1*, or *Vav* (Fig. S1, A and B). Such genetic interactions are not observed between *Epac* and *Abl* or *Rap1*, suggesting that Abl, Gef26, Rap1, and Vav, but not Epac, function together to mediate Gbb-induced presynaptic macropinocytosis. We therefore next examined genetic interactions in the regulation of the NMJ synaptic architecture.

After demonstrating the presynaptic requirement of Rap1 for normal regulation of synaptic architecture (Fig. 3, C and D), we next tested transheterozygous interactions among *Abl*, *Gef26*, *Epac*, *Rap1*, and *Vav* on synaptic growth. We found that single heterozygotes of *Abl*, *Gef26*, *Rap1*, and *Vav* have no effect, whereas transheterozygotes between *Abl* and *Gef26*, *Rap1*, or *Vav* show increases in total and satellite bouton numbers (Fig. S1, C and D). In contrast, synapses are normal in *Epac* and *Abl* or *Rap1* transheterozygotes. Thus, Abl, Gef26, Rap1, and Vav, but not Epac, function together to regulate synaptic growth.

**Figure 4.**
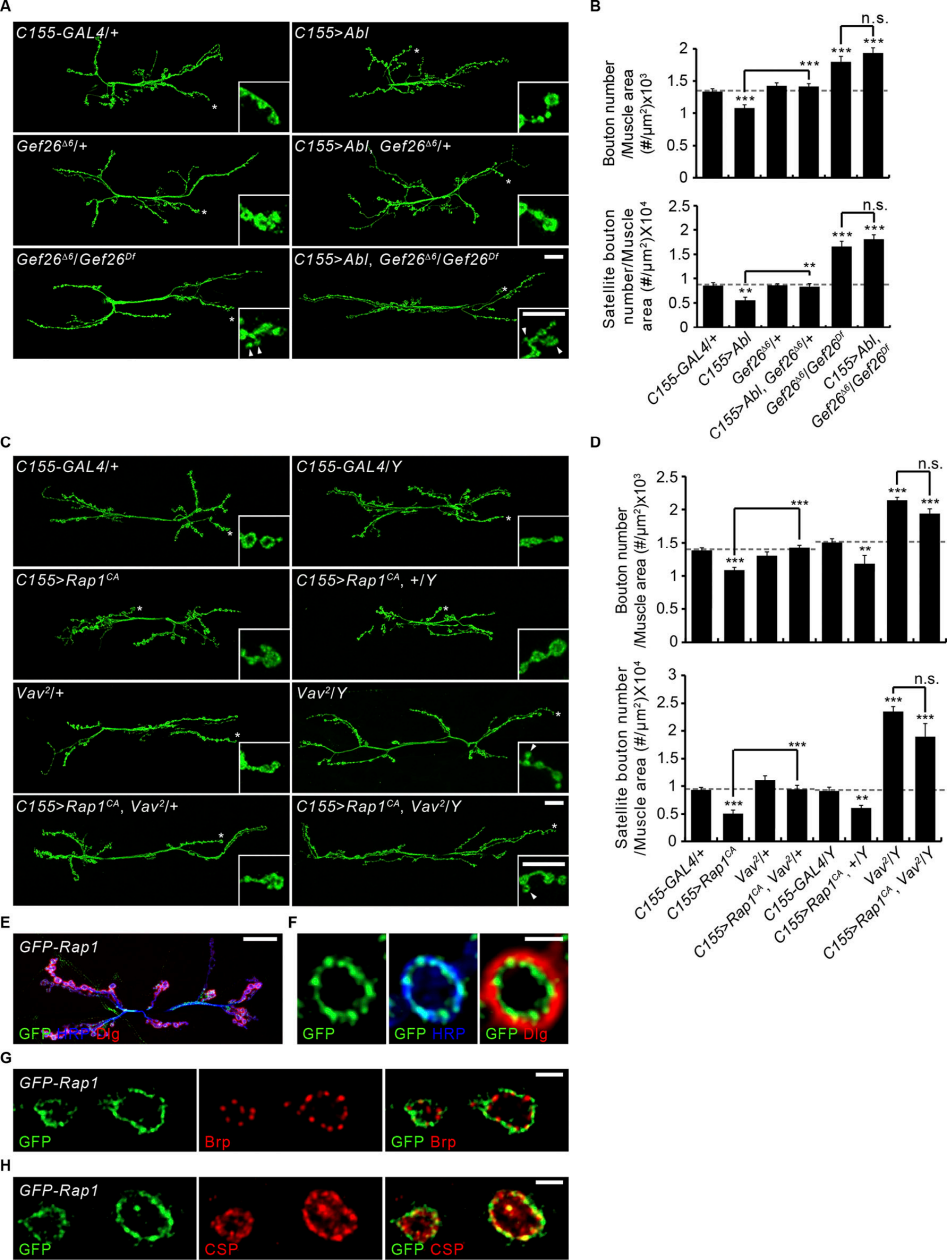
**Interaction of Gef26-Rap1 signaling and Abl/Vav during synaptic elaboration, and localization of Rap1 to NMJ presynaptic terminal. (A)** Representative NMJ 6/7 images of *C155-GAL4*/+, *C155-GAL4*/+; *UAS-Abl*/+ (*C155*>*Abl*), *Gef26*^*Δ6*^/+, *C155-GAL4*/+; *Gef26*^*Δ6*^,+/+,*UAS-Abl* (*C155*>*Abl*, *Gef26*^*Δ6*^/+), *Gef26*^*Δ6*^/*Gef26*^*Df*^, and *C155-GAL4*/+; *Gef26*^*Δ6*^,+/*Gef26*^*Df*^,*UAS-Abl* (*C155*>*Abl*, *Gef26*^*Δ6*^/*Gef26^Df^*) labeled for HRP. **(B)** Quantification of the total and satellite bouton numbers normalized to the muscle surface area. **(C)** NMJ images of *C155-GAL4*/+, *C155-GAL4*/+; *UAS-Rap1*^*CA*^/+ (*C155*>*Rap1*^*CA*^), *Vav*^*2*^/+, *Vav*^*2*^,*C155-GAL4*/+; *UAS-Rap1*^*CA*^/+ (*C155*>*Rap1*^*CA*^, *Vav*^*2*^/+), *C155-GAL4*/*Y*, *C155-GAL4*/*Y*; *UAS-Rap1*^*CA*^/+ (*C155*>*Rap1*^*CA*^, +/*Y*), *Vav*^*2*^/*Y*, and *Vav*^*2*^,*C155-GAL4*/*Y*; *UAS-Rap1*^*CA*^/+ (*C155*>*Rap1*^*CA*^, *Vav*^*2*^/*Y*). **(D)** Quantification of total and satellite bouton numbers normalized muscle surface area. *n* = 15 NMJs. **(E–H)** Presynaptic localization of Rap1 within the NMJ. **(E)** Confocal z-projection images of NMJ 6/7 in *Rap1* promoter*-GFP-Rap1* (*GFP-Rap1*) third instar larva triply labeled with anti-GFP, anti-HRP, and anti-DLG. **(F)** Single confocal slices of NMJ 6/7 in *GFP-Rap1* labeled with anti-GFP (green), anti-HRP (blue), and anti-DLG (red). **(G)** Single confocal slices of NMJ 6/7 in *GFP-Rap1* labeled with anti-GFP (green) and anti-Brp (red). **(H)** Single confocal slices of NMJ 6/7 in *GFP-Rap1* labeled with anti-GFP (green) and anti-CSP (red). Data represent mean ± SEM. Comparisons are with *C155-GAL4/+* and *C155-GAL4/Y* controls unless otherwise indicated (**, P < 0.01; ***, P < 0.001; n.s., not significant by one-way ANOVA with multiple comparisons). Scale bars: 20 μm (A, C, and E); 10 μm (A and C, inset); 2 μm (F–H).

Next, we tested the genetic hierarchy in *Gef26*-*Rap1* signaling with *Abl* or *Vav*. Neuronal Abl overexpression reduces the numbers of total and satellite boutons with complete suppression by removing one copy of *Gef26* (Fig. 4, A and B). Moreover, Abl overexpression phenotypes are further suppressed by removing both *Gef26* copies, with significant synaptic overgrowth. Phenotypes in *Gef26* mutants alone versus Abl-overexpressing *Gef26* do not differ significantly, suggesting Gef26 acts downstream of Abl. To test whether *Vav* is epistatic to *Rap1*, one copy of *Vav* was removed. This completely suppresses the synaptic undergrowth associated with neuronal overexpression of Rap1^CA^, a constitutively active form of Rap1 (Fig. 4, C and D). Complete loss of *Vav* further suppresses Rap1^CA^ overexpression phenotypes. Synaptic bouton numbers in *Vav* hemizygotes overexpressing Rap1^CA^ and *Vav* hemizygotes are not significantly different, suggesting Rap1 acts upstream of Vav. These findings suggest that Abl-Gef26-Rap1 signaling acts upstream of Vav to mediate Gbb-induced presynaptic macropinocytosis and limit Gbb-dependent synaptic growth.

Having demonstrated the presynaptic requirement for Rap1 in the regulation of macropinocytosis and synaptic growth (Heo et al., 2017), we next examined the distribution of Rap1 at the NMJ by immunohistochemistry. We used a functional *GFP-Rap1* transgene that is expressed under the control of the endogenous *Rap1* promoter (Knox and Brown, 2002). Anti-GFP labeling reveals strong expression at type I NMJs (Fig. 4 E). Importantly, GFP-Rap1 signals largely overlap the neuronal membrane marker HRP, but not the postsynaptic marker Discs large (Dlg; Fig. 4 F). At the presynaptic membrane, GFP-Rap1 signals do not overlap with the active zone marker Bruchpilot/NC82 but rather are distributed to distinctive areas surrounding active zones (Fig. 4 G). A small portion of the GFP-Rap1 signal is detected within presynaptic terminals as punctate structures that often overlap with the SV marker, cysteine-string protein (CSP; Fig. 4 H). Thus, the distribution of Rap1 protein at the NMJ is consistent with its role in presynaptic macropinocytosis.

### Rap1 acts together with Vav in tetanic vesicle mobilization and synaptic potentiation

**Figure 5.**
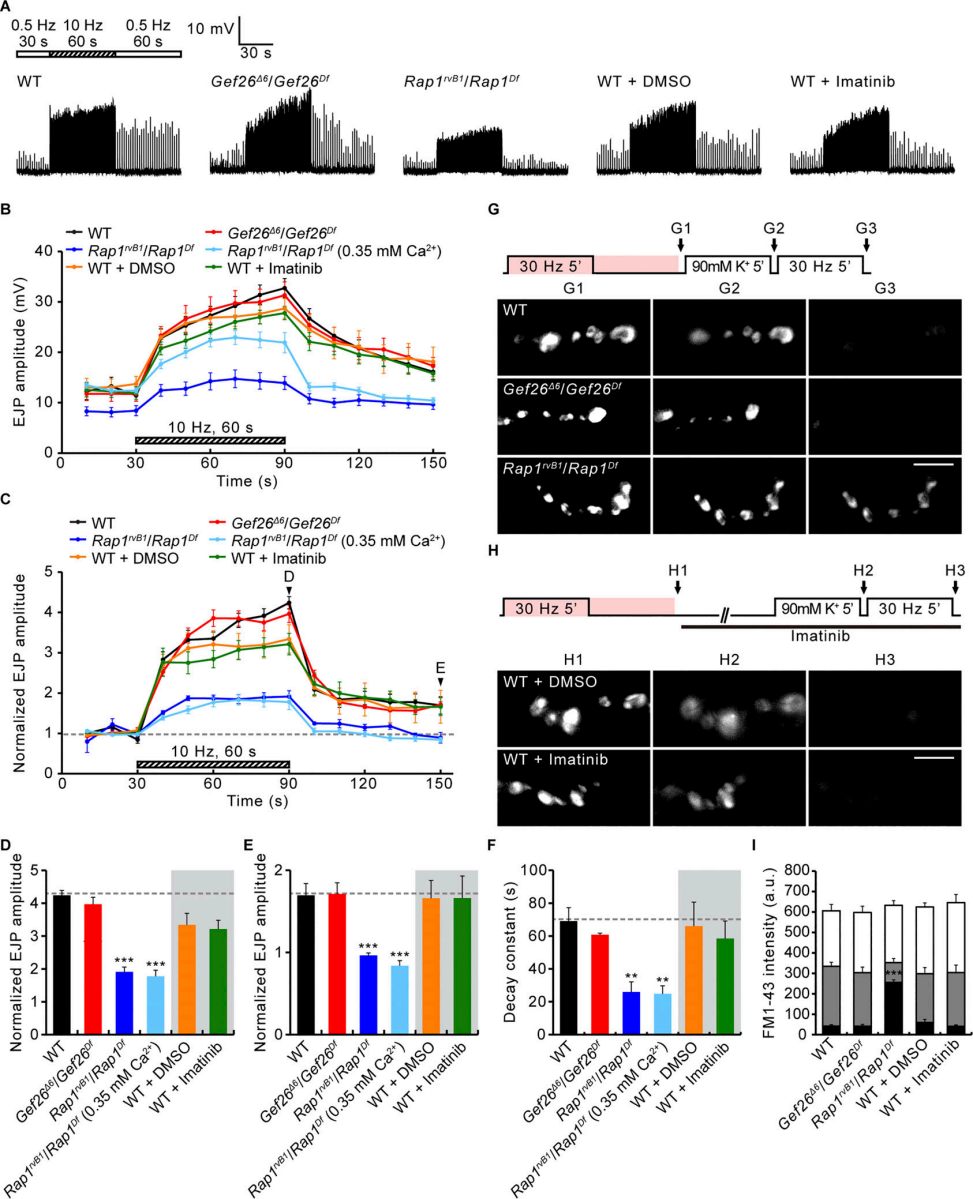
**Rap1, but not Abl or Gef26, mediates PTP and tetanus-induced RP mobilization. (A)** Representative recordings (muscle 6) of WT, *Gef26*^*Δ6*^/*Gef26*^*Df*^, and *Rap1*^*rvB1*^/*Rap1*^*Df*^ third instars in 0.3 mM Ca^2+^ saline stimulated at 0.5 Hz for 30 s (white bar), 10 Hz for 60 s (hatched bar), and then 0.5 Hz (white bar). Indicated preparations were incubated with 0.1% DMSO or imatinib (50 μM in 0.1% DMSO) for 30 min prior to and during recording. **(B)** EJP amplitudes before, during, and after 10-Hz 60-s tetanus. Each point in the ordinate indicates the mean amplitude of consecutive EJPs recorded every 10 s. For *Rap1*^*rvB1*^/*Rap1*^*Df*^ larvae, PTP was additionally induced in 0.35 mM Ca^2+^ saline. **(C)** Mean EJP amplitudes normalized to the initial amplitude at 0.5 Hz (basal). **(D–F)** Bar graphs of mean normalized EJP amplitudes after tetanus (D) and 60 s post-tetanus (E), and PTP decay time constants (F). *n* = 12 NMJs. **(G)** WT, *Gef26*^*Δ6*^/*Gef26*^*Df*^, and *Rap1*^*rvB1*^/*Rap1*^*Df*^ NMJs stimulated at 30 Hz for 5 min (2 mM Ca^2+^) with FM1-43 (pink, 4 μM), and imaged after a further 5 min without stimulation (G1; ECP-RP loading). Loaded boutons were then stimulated with high K^+^ (90 mM) for 5 min and imaged (G2; ECP unloading). ECP-unloaded, RP-loaded boutons were stimulated at 30 Hz for 5 min and imaged (G3; RP unloading). **(H)** Control boutons were loaded with FM1-43 (protocol in G) and imaged (H1; ECP-RP loading), and then treated with/without imatinib (50 μM) for 30 min, stimulated in high K^+^ (90 mM) for 5 min and imaged (H2; ECP unloading). ECP-unloaded, RP-loaded boutons were stimulated at 30 Hz for 5 min and imaged (H3; RP unloading). **(I)** FM1-43 fluorescence intensity in boutons after ECP-RP loading (whole columns), ECP unloading (gray columns), and RP unloading (black columns). *n* = 12 boutons. Data represent mean ± SEM. a.u., arbitrary units. Comparisons are with WT or DMSO-treated WT (**, P < 0.01; ***, P < 0.001 by one-way ANOVA with multiple comparisons). Scale bars: 5 μm.

**Figure 6.**
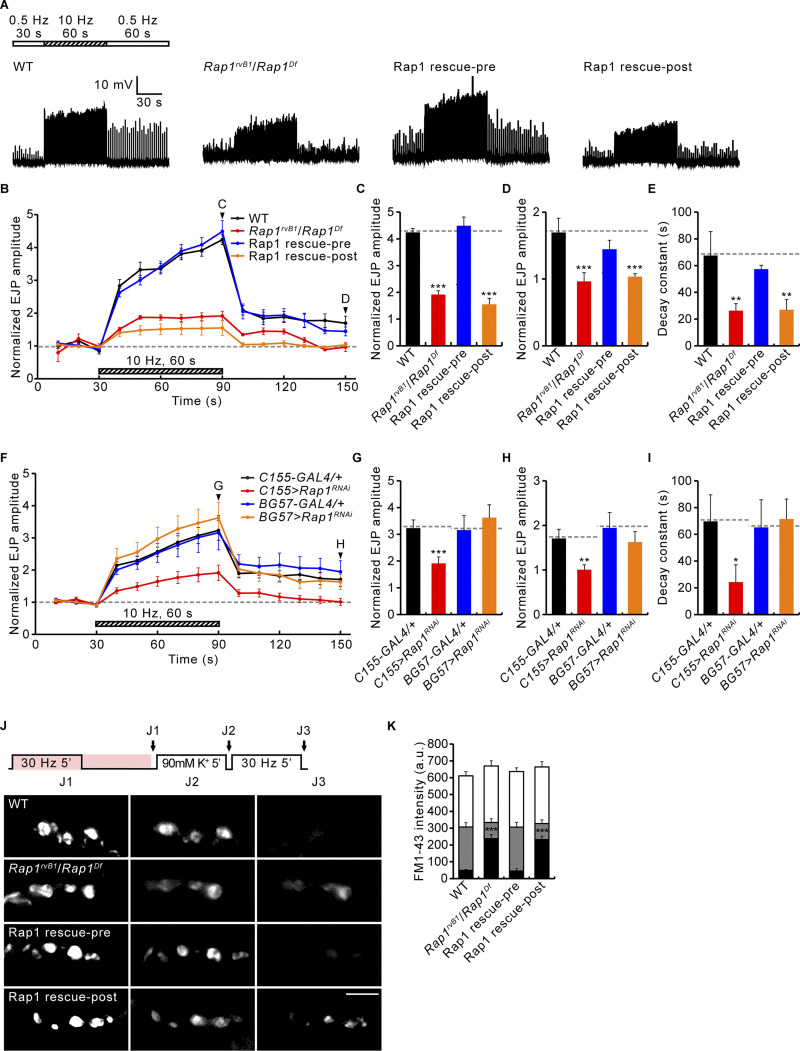
**Rap1 is required presynaptically for PTP and tetanus-induced RP mobilization. (A)** Representative recordings (muscle 6) of WT, *Rap1*^*rvB1*^/*Rap1*^*Df*^, *C155-GAL4*/+; *Rap1*^*rvB1*^,+/*Rap1*^*Df*^,*UAS*-*Rap1* (Rap1 rescue-pre); and *BG57-GAL4*,*Rap1*^*rvB1*^/*UAS-Rap1*,*Rap1*^*Df*^ (Rap1 rescue-post) third instar in 0.3 mM Ca^2+^. Stimulation was with 0.5 Hz for 30 s (white bar), 10 Hz for 60 s (hatched bar), and then 0.5 Hz (white bar). **(B)** Mean EJP amplitudes normalized to the initial amplitude at 0.5 Hz (basal). Each point indicates the mean normalized amplitude of consecutive EJPs recorded every 10 s. **(C–E)** Bar graphs of mean normalized EJP amplitudes after tetanus (C) and 60 s post-tetanus (D), and PTP decay time constants (E). *n* = 12 NMJs. **(F)** Mean EJP amplitudes for *C155-GAL4*/+, *C155-GAL4*/+; *UAS-Rap1*^*RNAi*^/+ (*C155*>*Rap1*^*RNAi*^), *BG57-GAL4*/+, and *BG57-GAL4*/+; *UAS-Rap1*^*RNAi*^/+ (*BG57*>*Rap1*^*RNAi*^) third instar larvae stimulated as in A. **(G–I)** Bar graphs of mean normalized EJP amplitudes after tetanus (G) and 60 s post-tetanus (H), and PTP decay time constants (I). *n* = 12 NMJs. **(J)** WT, *Rap1*^*rvB1*^/*Rap1*^*Df*^, *C155-GAL4*/+; *Rap1*^*rvB1*^,+/*Rap1*^*Df*^,*UAS*-*Rap1* (Rap1 rescue-pre), and *BG57-GAL4*.*Rap1*^*rvB1*^/*UAS-Rap1*,*Rap1*^*Df*^ (Rap1 rescue-post) NMJs stimulated at 30 Hz for 5 min (2 mM Ca^2+^) with FM 1–43 (pink, 4 μM), and imaged after a further 5 min without stimulation (J1; ECP-RP loading). Loaded boutons were then stimulated with high K^+^ (90 mM) for 5 min and imaged (J2; ECP unloading). Finally, ECP-unloaded, RP-loaded boutons were stimulated at 30 Hz for 5 min and imaged (J3; RP unloading). **(K)** FM1-43 fluorescence intensity in boutons after ECP-RP loading (height of whole columns), ECP unloading (height of gray columns), and RP unloading (height of black columns). Data represent mean ± SEM. *n* = 12 boutons. a.u., arbitrary units. Comparisons are with wild type (*, P < 0.05; **, P < 0.01; ***, P < 0.001 by one-way ANOVA with multiple comparisons). Scale bar: 5 μm.

In addition to Gbb-induced presynaptic macropinocytosis, Vav-Rac1-SCAR signaling likewise mediates PTP at the *Drosophila* NMJ (Park et al., 2022). To test whether the Abl-Gef26-Rap1 pathway is involved in PTP plasticity, the motor nerve was stimulated in 0.3 mM Ca^2+^ at a basal frequency (0.5 Hz) for 30 s, followed by tetanic stimulation at 10 Hz for 60 s, and then basal stimulation again at 0.5 Hz to monitor PTP. Initial analysis of excitatory junctional potential (EJP) amplitudes reveals that Rap1, but not Abl and Gef26, is specifically required for synaptic augmentation and PTP (Fig. 5, A and B). When we normalize EJP response to the initial level, WT NMJs display rapid facilitation, leading to a 324 ± 15% augmentation at the end of the tetanic stimulation, as well as PTP of 69 ± 21% over initial EJP amplitude at 60 s after tetanus (Fig. 5, C–E). The PTP phase of control NMJs is fitted with a monoexponential function with a decay time constant of 69.02 ± 8.22 s (Fig. 5 F). Wild-type NMJs pretreated with 50 μM imatinib or *Gef26* mutant NMJs show totally normal augmentation and PTP compared with the respective controls (Fig. 5, C–F), indicating that Abl and Gef26 do not play roles in these forms of short-term plasticity. By contrast, *Rap1* mutants show severely impaired augmentation and PTP. In *Rap1* mutants, EJP amplitudes are increased only 91 ± 14% at the end of tetanic stimulation and then slightly reduced to −11 ± 13% of initial value at 60 s after tetanus (Fig. 5, C–E). Moreover, the PTP decay time constant is also strongly reduced to 25.94 ± 6.14 s (Fig. 5 F). Notably, the pretetanic EJPs in *Rap1* mutants were significantly smaller than normal (Fig. 5 B; see Fig. S3, A and B for quantification). We, therefore, repeated PTP recording from *Rap1* mutants in 0.35 mM external Ca^2+^, at which *Rap1* pretetanic EJP amplitude is comparable with the WT amplitude in 0.3 mM Ca^2+^ (Fig. 5 B). Under this condition, *Rap1* mutants still show strongly reduced augmentation and PTP (Fig. 5, C–F), implying that these defects of *Rap1* mutants are unlikely to be secondary consequences of impaired basal synaptic transmission. Presynaptic, but not postsynaptic, expression of *UAS-Rap1* in *Rap1* mutants restores synaptic augmentation and PTP to WT levels (Fig. 6, A–F). In addition, the impaired PTP phenotype of *Rap1* mutants is recapitulated by presynaptic, but not postsynaptic, expression of *UAS-Rap1*^*RNAi*^ in the WT background (Fig. 6, F–I), confirming the presynaptic requirement for Rap1. Thus, in contrast to presynaptic macropinocytosis, PTP requires only presynaptic Rap1, but not Abl or Gef26.

Vav-Rac1-SCAR signaling mediates PTP by RP vesicle mobilization to the ECP (Park et al., 2022). We therefore tested the role of Rap1 in this mechanism. The lipophilic styrene dye FM1-43 was loaded into presynaptic boutons during (5 min, ECP loading) and after (5 min, RP loading) motor nerve stimulation at 30 Hz (Kuromi and Kidokoro, 2002). Fluorescence does not differ significantly between WT and *Gef26* or *Rap1* mutants or between untreated and imatinib-treated WT controls (Fig. 5, G–I), indicating that SV endocytosis does not require Abl, Gef26, or Rap1. To visualize loaded RP vesicles alone, ECP vesicles were unloaded by nerve stimulation with 90 mM K^+^ for 5 min. Fluorescence after stimulation is similar in all of the genotypes, indicating that ECP exocytosis is not impaired by Abl, Gef26, or Rap1 activity. To assess RP mobilization, 30 Hz stimulation was applied to ECP-unloaded boutons for 5 min. Following stimulation, FM1-43 fluorescence declined to background levels in *Gef26* mutants and imatinib-treated WT boutons (Fig. 5, G–I). In contrast, fluorescence in *Rap1* mutants is approximately sixfold higher than controls. Similar to the PTP defect, this impairment in RP vesicle mobilization is fully rescued by presynaptic, but not postsynaptic, expression of WT Rap1 (Fig. 6, J and K). These findings indicate that tetanic stimulation-induced RP mobilization is dependent on presynaptic Rap1, but not Abl or Gef26 function.

**Figure S2.**
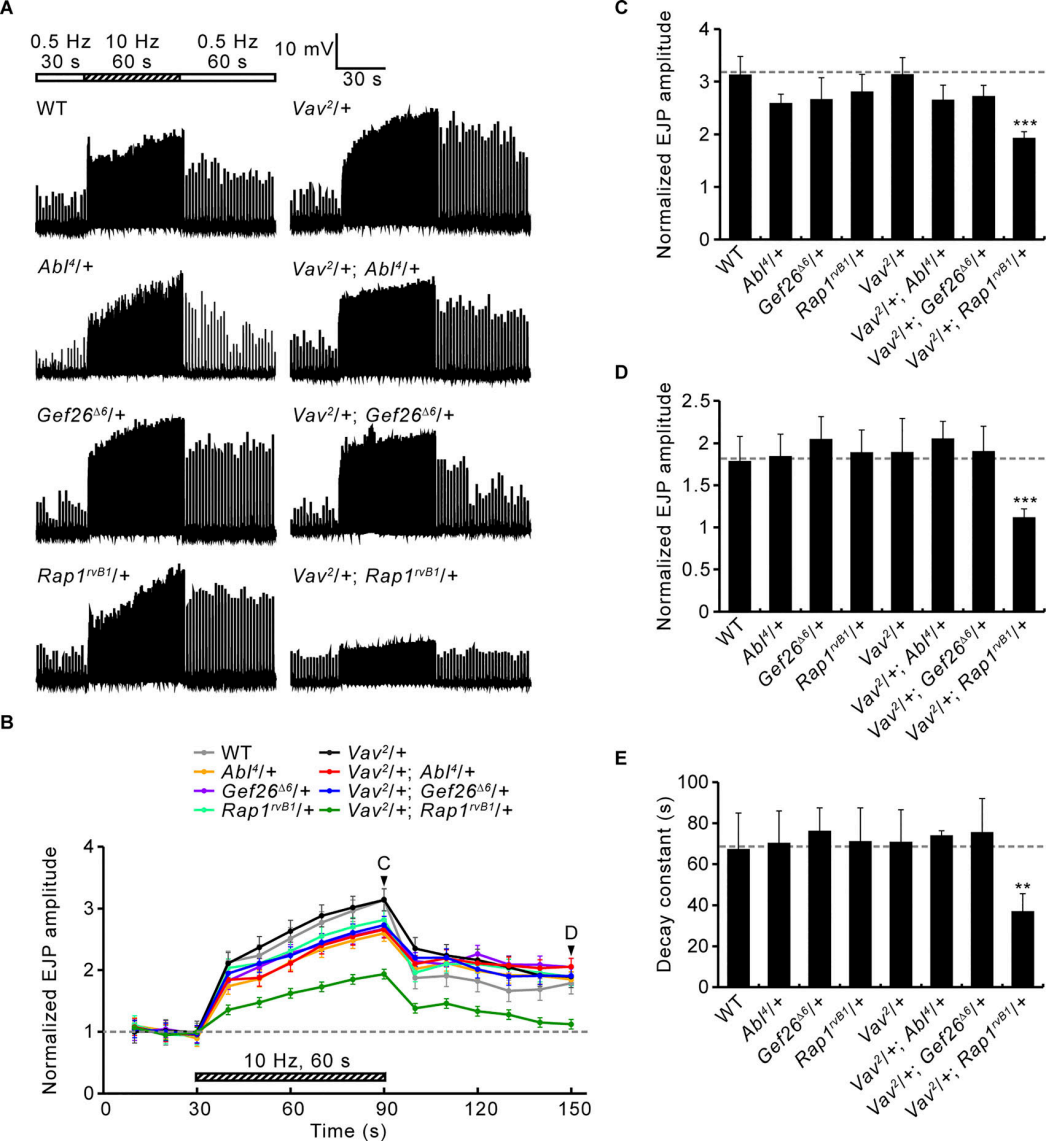
**Transheterozygous interactions between *Vav* and *Abl*, *Gef26*, or *Rap1* during PTP induction. (A)** Representative recordings (muscle 6) from third instar larvae of the indicated genotypes before, during, and after the 10 Hz, 1 min PTP induction protocol (0.3 mM Ca^2+^). **(B)** Mean EJP amplitudes normalized to the initial mean EJP amplitude at 0.5 Hz (basal). Each point indicates the mean normalized amplitude of consecutive EJPs recorded every 10 s. **(C–E)** Bar graphs of mean normalized EJP amplitudes after tetanus (C) and 60 s post-tetanus (D), and PTP decay time constants (E). *n* = 12 NMJs. Data represent mean ± SEM. Comparisons are with wild type (**, P < 0.01; ***, P < 0.001 by one-way ANOVA with multiple comparisons).

To confirm these exciting findings, we tested transheterozygous interactions of *Vav* with *Abl*, *Gef26*, or *Rap1* during presynaptic plasticity (Fig. S2). Animals carrying one null copy each of *Vav* and *Rap1* show strongly impaired synaptic augmentation and PTP, whereas these impairments were not observed in either of the single heterozygotes. In addition, the decay time constant is significantly reduced in *Vav* and *Rap1* transheterozygotes. These defects are also not present in animals transheterozygous for *Vav* and *Abl* or *Gef26*. These findings suggest that only Rap1 works together with Vav during the induction of synaptic augmentation and PTP, consistent with a model in which Abl and Gef26 do not contribute to these two forms of short-term plasticity.

**Figure S3.**
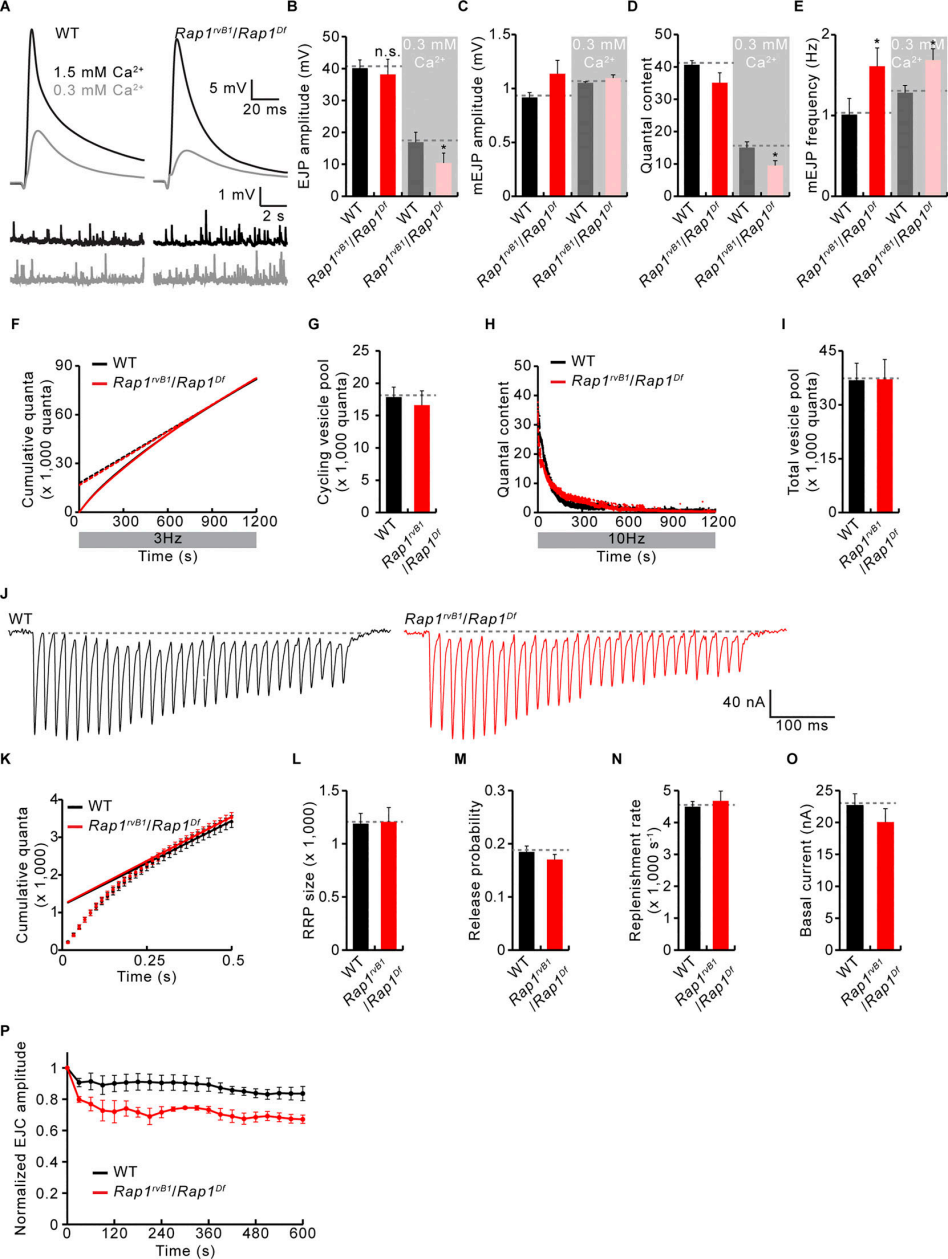
**Functional synaptic properties in *Rap1* mutants. (A–E)** Basal synaptic transmission in *Rap1* mutants. **(A)** Representative traces of evoked EJPs (0.5 Hz nerve stimulation) and spontaneous mEJPs from WT and *Rap1*^*rvB1*^*/Rap1*^*Df*^ larvae in 0.3 or 1.5 mM Ca^2+^ saline. **(B–E)** Quantifications of EJP amplitude (B), mEJP amplitude (C), quantal content (D) calculated by dividing the mean EJP amplitude by the mean mEJP amplitude, and mEJP frequency (E). *n* = 8 NMJs. **(F–I)** WT and *Rap1*^*rvB1*^*/Rap1*^*Df*^ third instar NMJs were continuously stimulated at 3 Hz (for analysis of ECP size) or 10 Hz (for analysis of total vesicle pool size) in the presence of 1 μM folimycin and 100 μM dynasore to block recycling of transmitter-containing synaptic vesicles. **(F)** Cumulative quantal release during 3 Hz stimulation. A-line fit to data points in a range of 900–1,200 s was back-extrapolated to time 0 to estimate ECP size (y-intercept). **(G)** Quantification of mean ECP sizes. **(H)** Cumulative quantal release during 10 Hz stimulation. **(I)** Quantification of total vesicle pool sizes as estimated by integrating quantal content over a 1,200 s period. **(J–O)** WT and *Rap1*^*rvB1*^*/Rap1*^*Df*^ larvae were subject to a 60 Hz train of 30 stimuli in 2 mM Ca^2+^ saline. *n* = 8 NMJs. **(J)** Representative traces of evoked EJCs. **(K)** Mean cumulative quantal content over time. A line fit to stimuli 18–30 was back-extrapolated to time 0 to estimate RRP size (y-intercept). **(L)** Quantification of mean RRP sizes. **(M)** Quantification of vesicular release probability estimated by dividing the first EJC amplitude by RRP size. **(N)** Quantification of RRP replenishment rate estimated from the slope of the line fit in J. **(O)** Quantification of basal current during the 60 Hz stimulation. **(P)** Plot of mean EJC amplitudes normalized to mean initial amplitude for WT and *Rap1*^*rvB1*^*/Rap1*^*Df*^ larvae during 10 Hz stimulation in 2 mM Ca^2+^ saline. *n* = 12 NMJs. Data represent mean ± SEM. *, P < 0.05 by two-sided unpaired Student’s *t* test.

Having demonstrated the roles of Rap1 in RP mobilization and PTP, we characterized other functional synaptic properties in *Rap1* mutants. When the motor nerve is stimulated at a low, basal frequency (0.5 Hz) in physiological saline (1.5 mM external Ca^2+^), the mean amplitudes of EJPs and miniature EJPs (mEJPs), and the mean evoked quantal content are not significantly altered in *Rap1* mutants compared with WT controls (Fig. S3, A–D). However, under PTP-inducing conditions (0.3 mM Ca^2+^), EJP amplitude and quantal content (QC), but not mEJP amplitude, are significantly lowered in *Rap1* mutants (Fig. S3, A–D). At both external Ca^2+^ concentrations, the frequency of miniature events is significantly increased in *Rap1* mutants (Fig. S3 E). The sizes of presynaptic vesicle pools including the readily releasable pool (RRP), ECP, and total pool are all normal in *Rap1* mutants (Fig. S3, F–L). Likewise, the vesicular release probability (*P*_*r*_), RRP replenishment rate, and basal current are also within normal ranges in *Rap1* mutants (Fig. S3, M–O). Finally, we characterized the ability of *Rap1* mutants to maintain synaptic transmission during high activity levels. At the end of 10-Hz stimulation in 2 mM Ca^2+^, WT NMJs are able to maintain EJC amplitudes at ∼83.6% of the initial response (Fig. S3 P). However, *Rap1* NMJs show enhanced depression to ∼67.2% of initial EJC amplitudes. Given that the RP sustains high-frequency transmission (Kuromi and Kidokoro, 1998; Verstreken et al., 2005), as well as SV endocytosis being normal in *Rap1* mutants (Fig. 5, G–I), the *Rap1* phenotype is consistent with a specific RP mobilization defect.

### Adenylate cyclase and cAMP-Epac signaling mediate PTP

**Figure 7.**
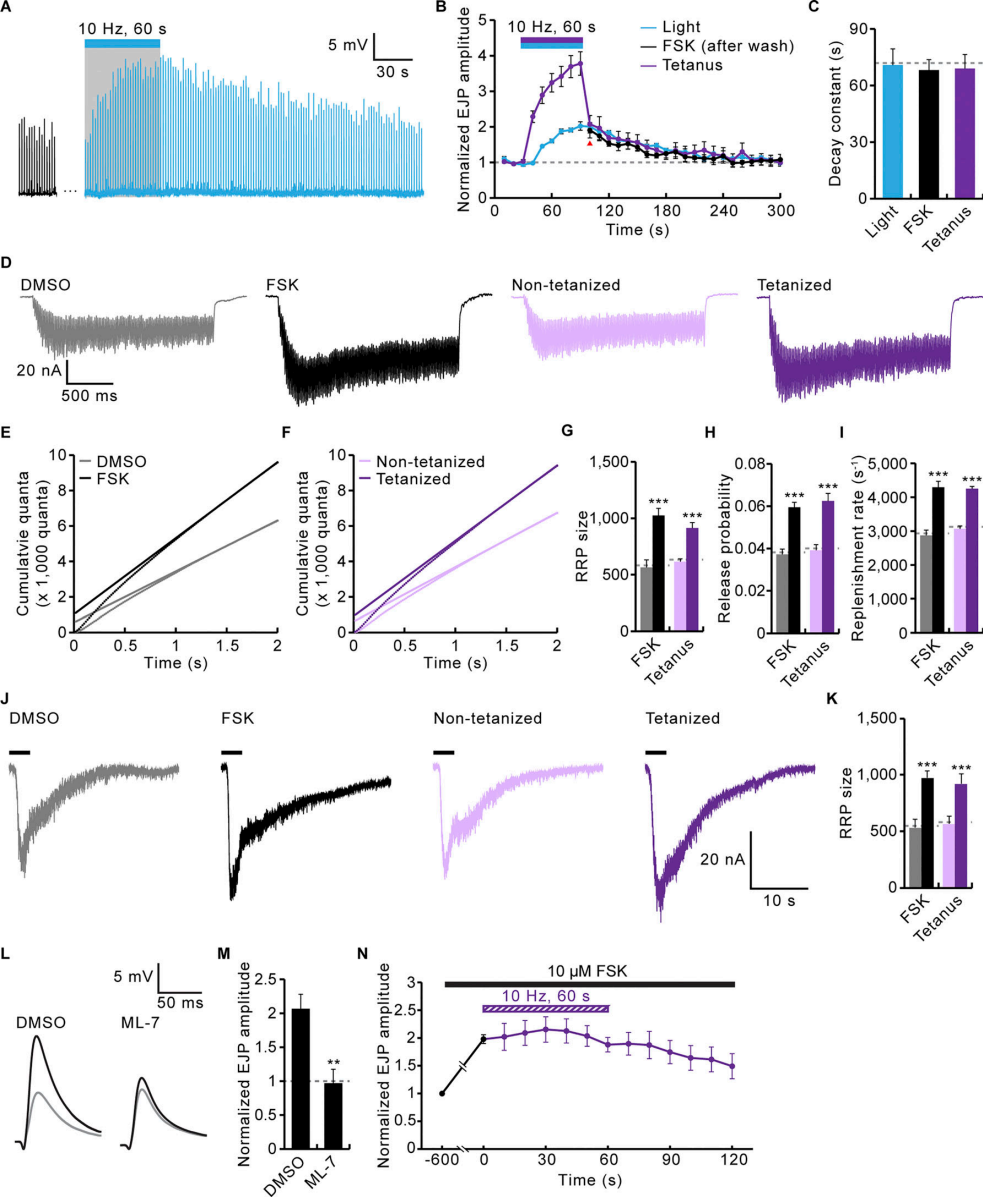
**Functional interactions between cAMP-induced synaptic potentiation and PTP. (A)** Representative recordings of *nSyb-GAL4*,+/+,*UAS-PACα* before, during, and after blue light stimulation (10 Hz, 60 s) of the photoactivable adenylate cyclase *PACα*. **(B)** Time courses of EJP potentiation (0.3 mM Ca^2+^) by blue light stimulation (*nSyb-GAL4*,+/+,*UAS-PACα*; blue), FSK (WT; 10 μM, 10 min; black), and PTP-inducing tetanus (WT; 10 Hz, 60 s; purple). Each point indicates the mean normalized amplitude of consecutive EJPs recorded every 10 s. **(C)** Decay time constants for EJP potentiation by PACα, FSK, and PTP-inducing tetanus. *n* = 8 NMJs. **(D–I)** Comparison of the effects of FSK and PTP-inducing tetanus on RRP size, vesicular *P*_*r*_, and RRP replenishment rate. **(D)** Representative EJC recording during a 60 Hz train (2 s, 0.3 mM Ca^2+^) from previously 0.1% DMSO/FSK-treated (gray/black) or non-tetanized/tetanized (pale purple/dark purple) WT larval preparations. **(E–G)** Quantal release over time is shown for DMSO/FSK-treated (E) or non-tetanized/tetanized (F) preparations, with an extrapolated fitted line to t = 0 to estimate RRP size (y-intercept) reported in G. **(H)** Vesicular *P*_*r*_ determined by dividing initial EJP amplitude by RRP size. **(I)** RRP replenishment rate determined from the regression line slope. *n* = 8 NMJs. **(J and K)** Sucrose estimates of RRP sizes. **(J)** Recordings from 0.1% DMSO/FSK-treated (gray/black) or non-tetanized/tetanized (pale purple/dark purple) WT larvae using hypertonic sucrose (500 mM for 3 s, black bar). **(K)** Quantification of RRP size, as determined by the total sucrose charge divided by the mEJC charge. **(L and M)** The effect of the MLCK inhibitor ML-7 on FSK-induced potentiation of EJPs. **(L)** Representative EJP recordings from WT larval preparations before (gray) and after (black) FSK stimulation (10 μM, 10 min). NMJs had been incubated in 0.1% DMSO with or without 15 μM ML-7 for 30 min. **(M)** Quantification of FSK potentiation. *n* = 12 NMJs. **(N)** Normalized EJP amplitudes in WT preparations treated with 10 μM FSK for 10 min (black) and subsequently subjected to a 10 Hz, 60 s train in the continued presence of FSK (purple). Each point indicates the mean normalized amplitude of consecutive EJPs recorded every 10 s. *n* = 8 NMJs. Data represent mean ± SEM. **, P < 0.01; ***, P < 0.001 (C, G–I, and K: one-way ANOVA with multiple comparisons; M: two-sided unpaired Student’s *t* test).

FSK-induced elevation of cAMP is sufficient to enhance both synaptic transmission strength and RP vesicle mobilization (Cheung et al., 2006; Kuromi and Kidokoro, 2000), mimicking the effects of PTP-inducing tetanus. Furthermore, the adenylate cyclase Rutabaga (Rut) is absolutely required for tetanus-induced RP mobilization and PTP (Kuromi and Kidokoro, 2000; Zhong and Wu, 1991). To test for the direct involvement of Rut-dependent cAMP signaling in the production of PTP, we assayed the possible mechanistic overlaps between the two processes. We initially compared decay time constants of cAMP-induced potentiation and PTP. cAMP elevation was triggered by the optogenetic activation of photoactivable adenylate cyclase (PACα; Schröder-Lang et al., 2007) as well as by FSK application (10 μM). Pan-neuronal activation of PACα by 10 Hz blue light pulses for 60 s significantly increases basal EJP amplitudes by 100 ± 6% over the initial amplitude, comparable with the 107 ± 25% increase in an early phase of PTP at 10 s after tetanic stimulation (Fig. 7, A and B). A similar level of synaptic potentiation (90 ± 21%) was observed at the washout phase after acute application of FSK (Fig. 7 B; red arrowhead). Importantly, synaptic potentiation by both PACα activation and FSK application persists for >2 min, with PACα (70.87 ± 8.58 s, P = 0.969) and FSK (68.37 ± 5.49 s, P = 0.968) having decay time constants similar to PTP (69.02 ± 8.22 s; Fig. 7 C). These findings suggest that the processes likely share a common mechanism.

We next compared the effects of FSK application and PTP-inducing tetanus on RRP size, vesicular *P*_*r*_, and RRP replenishment rate. We used cumulative postsynaptic current analyses during high-frequency stimulus trains (60 Hz, 2 s) in 0.3 mM Ca^2+^ saline (Müller et al., 2012). FSK treatment and PTP-inducing tetanus similarly increase RRP size (81 ± 27% versus 77 ± 7%), vesicular *P*_*r*_ (60 ± 23% versus 72 ± 11%), and replenishment rate (49 ± 12% versus 39 ± 2%) (Fig. 7, D–I). The effects of FSK and PTP-inducing tetanus on RRP size were independently tested by hypertonic sucrose challenge in calcium-free saline. The sucrose-sensitive synaptic vesicle pool is significantly increased by prior application of FSK or PTP-inducing tetanus (83 ± 12% versus 63 ± 16%; Fig. 7, J and K), confirming that both FSK-induced potentiation and PTP are paralleled with an increase in RRP size.

ML-7 is an inhibitor of myosin light chain kinase (MLCK) that impairs PTP by interfering with RP vesicle mobilization (Kim et al., 2009; Park et al., 2022). We therefore next tested the effects of ML-7 application on the FSK-induced synaptic potentiation. Compared with the vehicle-only control (DMSO), FSK application reliably increases EJP amplitude by 107 ± 21%, but the prior application of the ML-7 inhibitor completely blocks the FSK-dependent potentiation of neurotransmission strength (Fig. 7, L and M). Moreover, we performed occlusion experiments to determine whether FSK potentiation and PTP interact with one another. Prior FSK application (10 μM, 10 min) completely inhibits the subsequent induction of PTP (Fig. 7 N), implying that FSK potentiation and PTP share a common process. Combined with results showing that the Rut is required for tetanus-induced RP mobilization and PTP (Kuromi and Kidokoro, 2000; Zhong and Wu, 1991), these mechanistic overlaps strongly support the model in which Rut-dependent cAMP signaling plays a direct obligatory role in the induction of PTP in a presynaptic vesicle trafficking mechanism.

**Figure 8.**
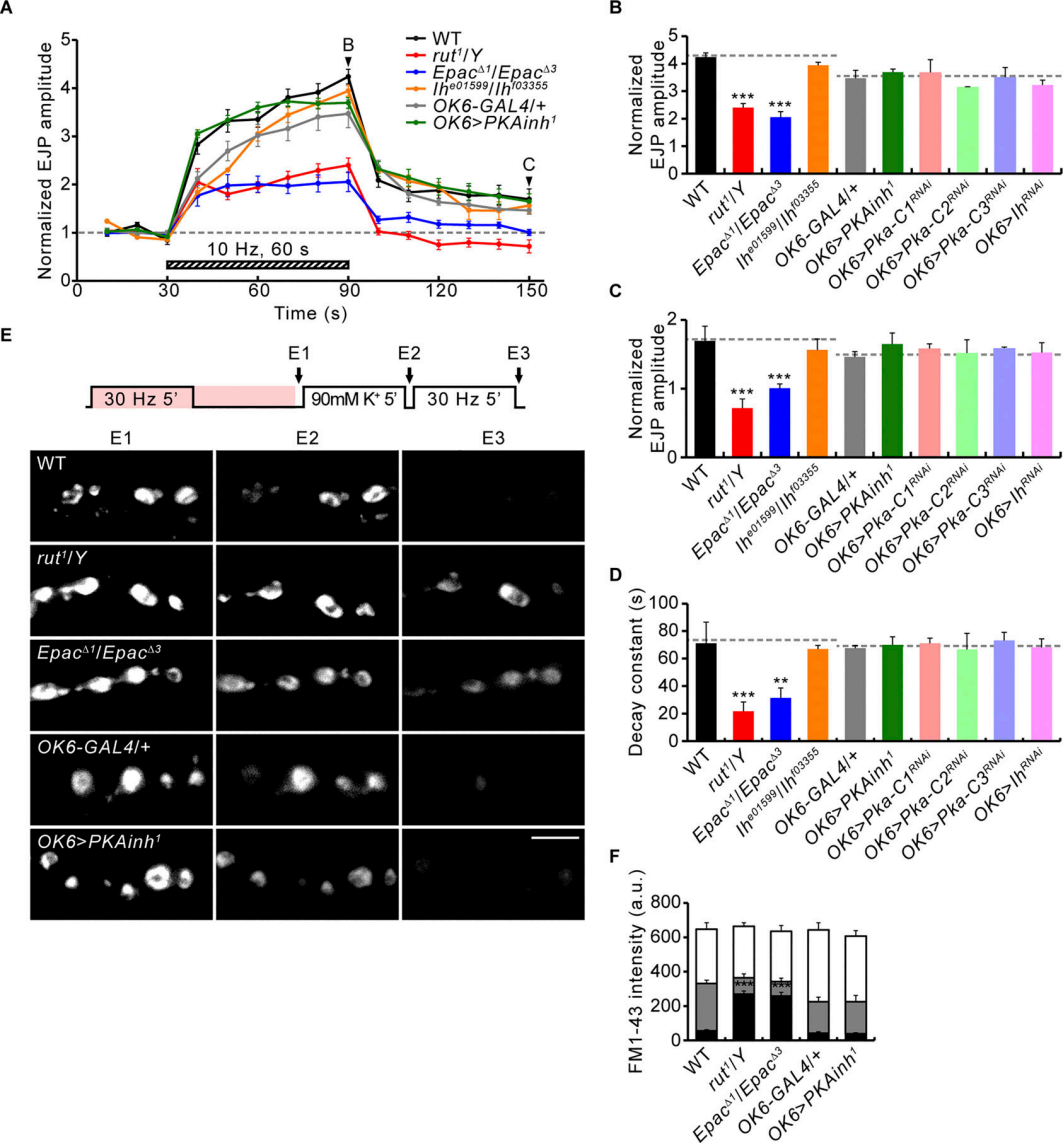
**Loss of Rut or Epac, but not PKA or the I**_**h**_ **channel, results in defective PTP. (A)** Representative recordings of WT, *rut*^*1*^/*Y*, *Epac*^*Δ1*^/*Epac*^*Δ3*^, *I*_*h*_^*e0159*9^/*I*_*h*_^*f03355*^, *OK6-GAL4*/+, and *OK6*-*GAL4*/*UAS*-*PKAinh*^*1*^ (*OK6>PKAinh*^*1*^) before, during, and after the 10 Hz, 1 min PTP induction protocol (0.3 mM Ca^2+^; Fig. 5 A). Mean EJP amplitudes normalized to the initial EJP amplitude at 0.5 Hz (basal). Each point indicates the mean normalized amplitude of consecutive EJPs recorded every 10 s. **(B–D)** Bar graphs of EJP amplitudes in larvae of indicated genotypes at the end (B) and 60 s after (C) tetanic stimulation, and PTP decay time constants (D). *n* = 12 NMJs. **(E)** NMJs with FM1-43 (pink) after ECP-RP loading (E1), ECP unloading (E2), and RP unloading (E3; as in Fig. 5 F) shown for WT, *rut^1^*/*Y*, *Epac^^Δ^1^*/*Epac^Δ3^*, *OK6-GAL4*/+, and *OK6>PKAinh^1^*. **(F)** FM1-43 fluorescence intensity after ECP-RP loading (whole columns), ECP unloading (gray columns), and RP unloading (black columns). *n* = 12 boutons. Data represent mean ± SEM. Comparisons are with WT or *OK6-GAL4*/+ (**, P < 0.01; ***, P < 0.001 by one-way ANOVA with multiple comparisons). Scale bar: 5 μm.

**Figure S4.**
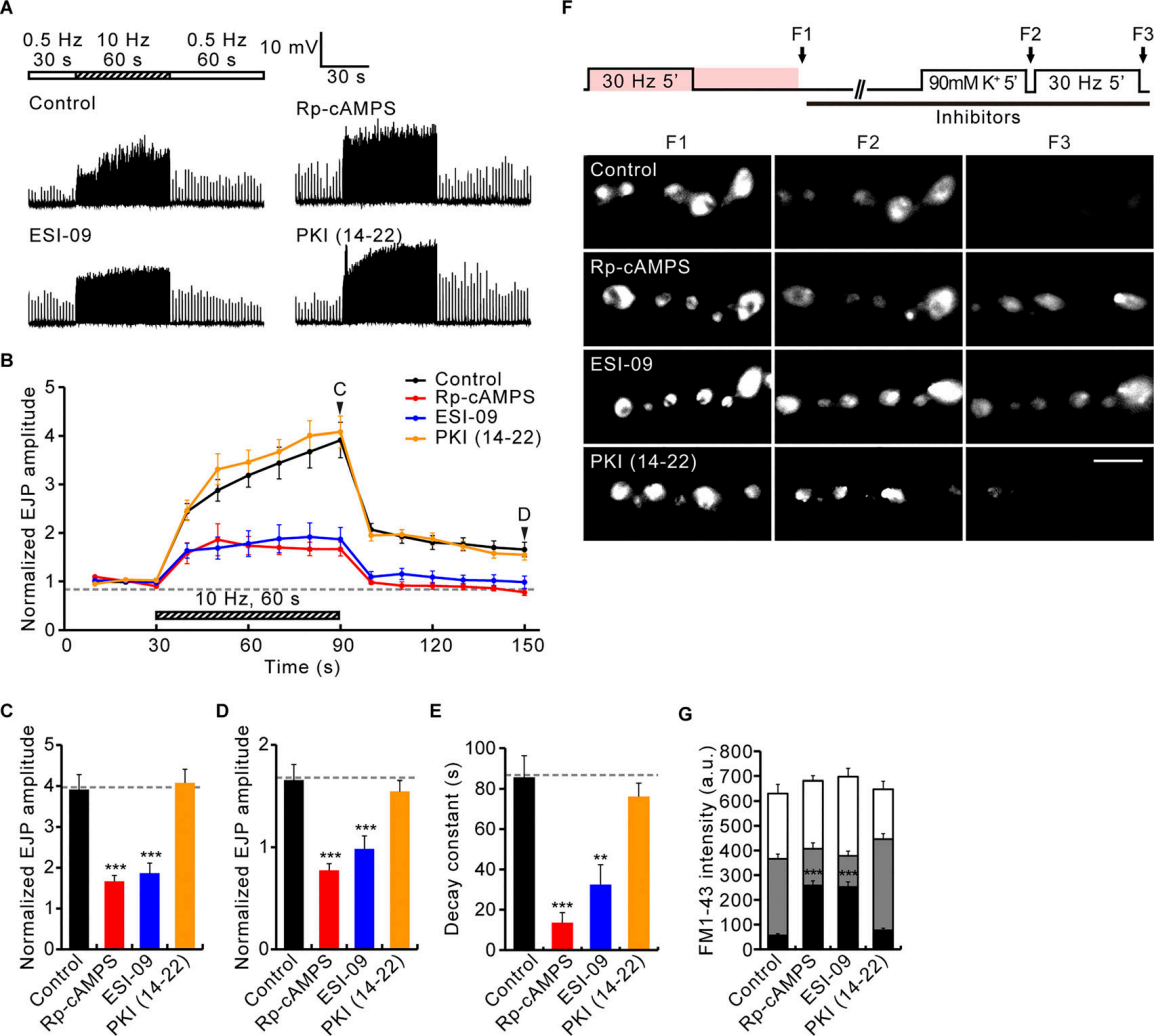
**Acute blockade of Epac, but not of PKA, impairs PTP and tetanus-induced RP mobilization. (A–D)** NMJ preparations of WT third instar larvae were incubated in 0.3 mM Ca^2+^ saline with 0.1% DMSO (control), 100 μM Rp-cAMPS (cAMP antagonist), or 20 μM ESI-09 (Epac inhibitor) for 20 min, or with 5 μM PKI-(14-22)-amide (PKA inhibitor) for 30 min, followed by PTP experiments in the same bath. **(A)** Representative EJP recordings from NMJ preparations before, during, and after a 10 Hz, 1 min PTP induction protocol. **(B)** Mean EJP amplitudes normalized to the initial mean EJP amplitude at 0.5 Hz (basal). Each point indicates the mean normalized amplitude of consecutive EJPs recorded every 10 s. **(C–E)** Bar graphs of mean normalized EJP amplitudes after tetanus (C) and 60 s post-tetanus (D), and PTP decay time constants (E). *n* = 12 NMJs. **(F)** Boutons in WT third instar larvae were stimulated at 30 Hz for 5 min in saline containing 2 mM Ca^2+^ and 4 μM FM1-43 (pink color), incubated in the same bath without stimulation for a further 5 min, washed with Ca^2+^-free saline, and imaged (F1; ECP-RP loading). Subsequently, loaded boutons were treated with 0.1% DMSO (control), 100 μM Rp-cAMPS, or 20 μM ESI-09 for 20 min, or with 5 μM PKI-(14-22)-amide for 30 min, incubated with 90 mM K^+^ for 5 min, and imaged (F2; ECP unloading). ECP-unloaded, RP-loaded boutons were subsequently stimulated at 30 Hz for 5 min and imaged (F3; RP unloading). **(G)** FM1-43 fluorescence intensity in boutons after ECP-RP loading (height of whole columns), ECP unloading (height of gray columns), and RP unloading (height of black columns). *n* = 12 boutons. Data represent mean ± SEM. a.u., arbitrary units. Comparisons are with DMSO-treated control (**, P < 0.01; ***, P < 0.001 by one-way ANOVA with multiple comparisons). Scale bar: 5 μm.

At invertebrate and vertebrate synapses, the cAMP-induced potentiation of synaptic transmission has been shown to be mediated by protein kinase A (PKA), Epac, and/or cyclic nucleotide-activated I_h_ channels (Beaumont and Zucker, 2000; Cheung et al., 2006; Fernandes et al., 2015; Gekel and Neher, 2008; Kaneko and Takahashi, 2004; Kuromi and Kidokoro, 2000; Weisskopf et al., 1994; Zhong and Zucker, 2005). To determine the downstream targets of cAMP signaling that drive PTP at the *Drosophila* NMJ, we tested the effects of *PKA*, *Epac*, or *I*_*h*_ loss of function on RP mobilization and PTP. Tetanus-induced RP mobilization and PTP are both strongly impaired in *rut* mutants (Fig. 8), as previously reported (Kuromi and Kidokoro, 2000; Zhong and Wu, 1991). Notably, loss of *Epac* recapitulates the phenotypes of *rut* mutants, whereas overexpression of a dominant-negative form of PKA regulatory subunit (PKAinh^1^) has no effect on RP mobilization or PTP (Fig. 8). Lack of a PKA role in PTP is further confirmed by motor neuron-specific knockdown of each of three PKA catalytic subunits (PKA-C1, -C2, and -C3; Fig. 8 B). Furthermore, *I*_*h*_ gene disruption or knockdown does not impair RP mobilization and PTP (Fig. 8). These findings indicate that Epac is a major target of Rut-dependent cAMP signaling in mediating presynaptic vesicle trafficking and PTP. This conclusion is confirmed by acutely blocking Epac or PKA using the cAMP signaling antagonist Rp-cAMPS (Christensen et al., 2003; Rehmann et al., 2003), the Epac-specific inhibitor ESI-09 (Almahariq et al., 2013), as well as the PKA-specific inhibitor PKI-(14-22)-amide (Dalton and Dewey, 2006). Application of PKI-(14-22)-amide (5 μM) does not affect RP vesicle mobilization or PTP, whereas application of Rp-cAMPS (100 μM) or ESI-09 (20 μM) severely impairs RP mobilization and abolishes PTP (Fig. S4). These findings indicate that Rut-dependent cAMP signaling acts through Epac to mediate presynaptic potentiation at the *Drosophila* NMJ.

### Rut-cAMP-Epac signaling acts through the Rap1-Vav to mediate PTP

**Figure 9.**
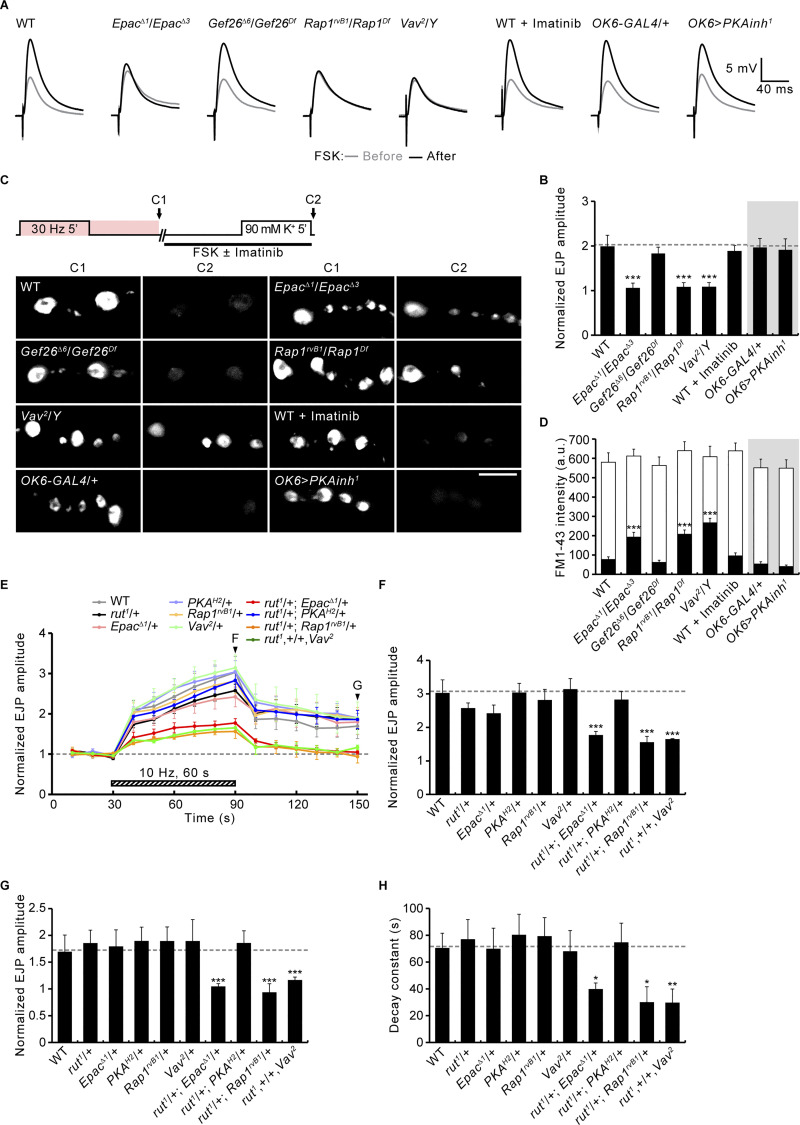
**PTP and RP mobilization by Rut-cAMP-Epac signaling acting via Rap1-Vav. (A)** Averaged EJP records from third instar larvae of indicated genotypes before (gray) and after (black) FSK stimulation (10 μM, 10 min). **(B)** Mean magnitudes of FSK-induced potentiation. *n* = 12 NMJs. **(C)** Diagram of FM1-43 ECP-RP loading/unloading protocol and representative NMJ bouton images after loading (C1) and unloading (C2). **(D)** FM1-43 fluorescence intensity in boutons before (C1, whole columns) and after (C2, black columns) K^+^-induced ECP unloading. *n* = 12 boutons. **(E)** Plot of EJP amplitudes normalized to initial in transheterozygous for *rut* and *Epac*, *PKA*, *Rap1*, or *Vav* with the PTP induction protocol described in Fig. 5 A. Each point indicates the mean normalized amplitude of consecutive EJPs recorded every 10 s. **(F–H)** Bar graphs of normalized EJP amplitudes at the end (F) and 60 s after (G) tetanic stimulation, and PTP decay time constants (H). *n* = 12 NMJs. Data represent mean ± SEM. Comparisons are with WT (*, P < 0.05; **, P < 0.01; ***, P < 0.001 by one-way ANOVA with multiple comparisons).

**Figure S5.**
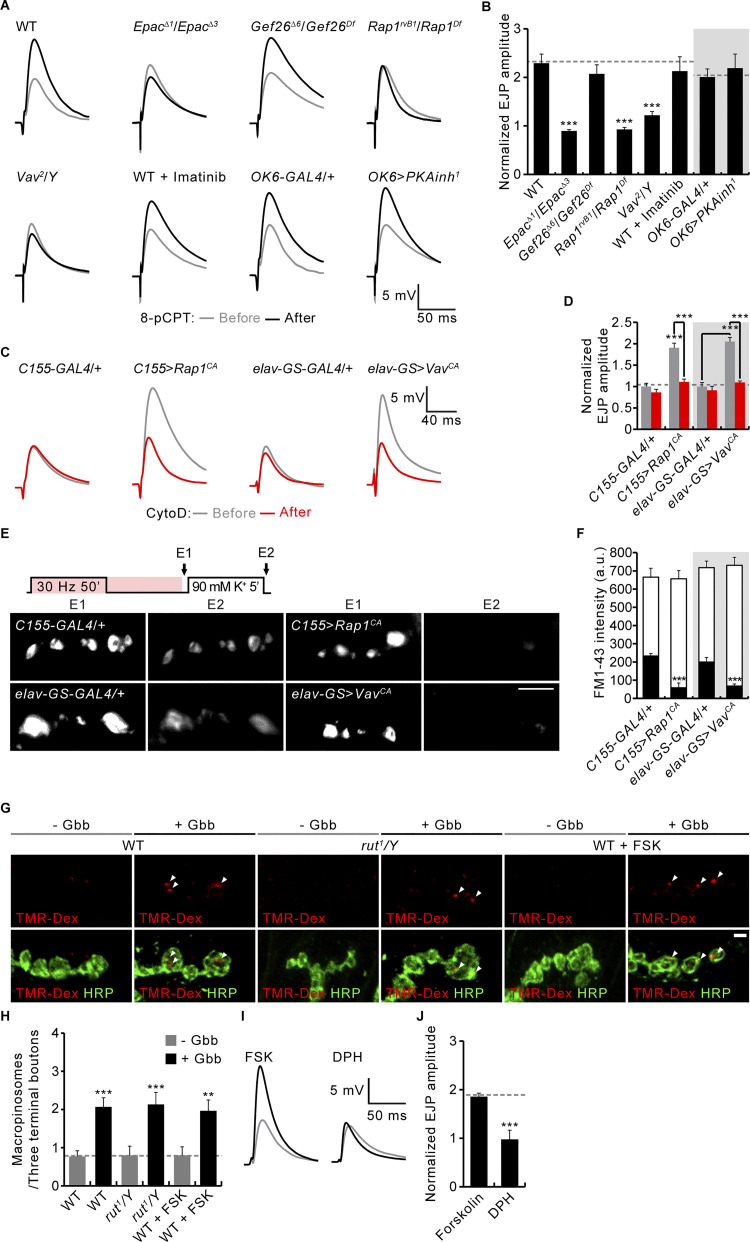
**Epac acts through the Rap1-Vav pathway to potentiate EJP amplitudes. (A)** Averaged sample records (from 15 events each) of EJPs from third instar NMJs of indicated genotypes before (gray) and after (black) 8-pCPT stimulation (100 μM, 30 min). **(B)** Mean magnitude of 8-pCPT-induced potentiation in third instar larvae of indicated genotypes. *n* = 12 NMJs. **(C)** Averaged sample records (from 15 events each) of EJPs from *C155-GAL4*/+, *C155-GAL4*/+; *UAS-Rap1*^*CA*^/+ (*C155*>*Rap1*^*CA*^), *elav-GS-GAL4*/+, and *elav-GS-GAL4*/*UAS-Vav*^*CA*^ (*elav-GS*>*Vav*^*CA*^) third instars before (gray) and after (black) application of CytoD (10 μM, 40 min), an inhibitor of actin polymerization. **(D)** Mean magnitude of EJP potentiation induced by Rap1^CA^ or Vav^CA^ overexpression before (gray) and after (red) CytoD treatment. *n* = 12 NMJs. **(E)** Diagram of FM1-43 loading/unloading protocol and representative images of NMJ boutons after ECP-RP loading by electrical stimulation at 30 Hz (E1) and ECP unloading by stimulation with 90 mM K^+^ (E2). **(F)** FM1-43 fluorescence intensity in boutons after ECP-RP loading (height of whole columns) and ECP unloading (height of black columns). *n* = 12 boutons. **(G)** Representative images of NMJ 6/7 terminals of WT third instar larvae labeled with membrane anti-HRP (green) following a 5-min pulse of TMR-Dex (red, 2 mg/ml) in the absence/presence of Gbb (50 ng/ml). Indicated preparations were pre-treated with DPH (10 μM) for 30 min. **(H)** Quantification of the number of TMR-Dex-positive puncta per three terminal boutons. **(I)** Averaged EJP records from WT third instar larvae before (gray) and after (black) FSK (10 μM, 10 min) or DPH (10 μM, 30 min) stimulation. **(J)** Mean magnitudes of FSK- and DPH-induced potentiation. *n* = 12 NMJs. Data represent mean ± SEM. a.u., arbitrary units. Comparisons are with an appropriate GAL4 control unless indicated. **, P < 0.01; ***, P < 0.001 (B, D, F, and H: one-way ANOVA with multiple comparisons; J: two-sided unpaired Student’s *t* test). Scale bars: 5 μm (E); 2 μm (G).

FSK and cAMP are both known to activate Rap1 through Epac proteins in mammalian cells (de Rooij et al., 1998; Kawasaki et al., 1998), suggesting that Rut-cAMP-Epac signaling may act through the Rap1-Vav pathway during presynaptic potentiation. This possibility was first tested by assessing whether Rap1 and Vav are required for synaptic potentiation induced by FSK or the Epac-specific cAMP analog 8-pCPT-2′-O-Me-cAMP (8-pCPT; Christensen et al., 2003). Bath application of FSK (10 μM) significantly increases evoked EJP amplitudes by 99 ± 25% (Fig. 9, A and B) but does not significantly affect the amplitudes of mEJPs (1.17 ± 0.11 mV versus 1.15 ± 0.08 mV; P = 0.23), suggesting that FSK acts on presynaptic release. FSK-induced EJP potentiation is strongly impaired in *Epac* (6 ± 10%), *Rap1* (8 ± 9%), and *Vav* (9 ± 9%) mutants (Fig. 9, A and B). Similar to FSK, 8-pCPT (100 μM) potentiates neurotransmission strength by 129 ± 19%, an effect blocked in *Epac* (−11 ± 3%), *Rap1* (−7 ± 4%), and *Vav* (22 ± 8%) mutants (Fig. S5, A and B). In contrast, the FSK or 8-pCPT response remains unaffected in *Gef26* mutants and by either presynaptic expression of PKAinh^1^ or prior application of imatinib (Fig. 9, A and B; and Fig. S5, A and B). These results strongly support the conclusion that Rut-cAMP-Epac signaling acts through the Rap1-Vav pathway to potentiate neurotransmitter release, independently of PKA, Abl, and Gef26.

The ability of FSK to mobilize RP vesicles to the ECP, even in the absence of tetanic stimulation (Kuromi and Kidokoro, 2000), suggests that FSK-induced RP vesicle mobilization may also require Epac and the Rap1-Vav pathway. WT boutons loaded with FM1-43 using the 30 Hz 5 min +5 min protocol (ECP-RP loading; Fig. 9 C) were subsequently treated with FSK application (10 μM) for 10 min. Stimulation of these FSK-treated animals with high K^+^ depolarization for 5 min to unload the ECP results in the loss of most presynaptic RP vesicle labeling (Fig. 9, C and D). In sharp contrast, FSK-untreated WT boutons retained much of the RP labeling after the high K^+^ unloading (see Fig. 5 G), confirming that FSK is able to induce RP mobilization to the ECP. Under the same conditions, FSK-treated *Epac*, *Rap1*, and *Vav* boutons retain most of their RP labeling after the high K^+^ unloading (Fig. 9, C and D). In contrast, the high K^+^ unloading of RP vesicles is not affected in *Gef26* mutants or by presynaptic expression of PKAinh^1^ or prior application of imatinib. These findings suggest that the Rap1-Vav pathway, but not PKA, Abl, and Gef26, acts to mediate FSK-induced, Epac-mediated RP mobilization. It was therefore critical to test whether Rap1 or Vav activation alone is sufficient to elevate synaptic transmission and induce RP mobilization in the absence of FSK stimulation.

Targeted neuronal expression of constitutively-active Rap1 (Rap1^CA^) or Vav (Vav^CA^) strongly potentiates neurotransmission strength by 90 ± 11% and 105 ± 29%, respectively (Fig. S5, C and D). Neither Rap1^CA^ nor Vav^CA^ affects the FM1-43 loading of total vesicles using the 30 Hz 5 min +5 min protocol, but both selectively reduce RP labeling after ECP unloading by high [K^+^] for 5 min (Fig. S5, E and F). Thus, presynaptic Rap1^CA^ and Vav^CA^ mimic the stimulatory effects of FSK on synaptic transmission and RP mobilization, supporting a functional link between the Rut-cAMP-Epac and Rap1-Vav pathways during PTP. The latter hypothesis was next tested directly by examining transheterozygous phenotypes of *rut* and *Epac*, *PKA-C1*(*PKA^H2^*), *Rap1*, or *Vav* mutants during and after tetanic stimulation. In animals heterozygous for each of these genes, levels of tetanus-induced synaptic augmentation and PTP are normal or only slightly reduced (P > 0.05; Fig. 9, E–G). Both forms of synaptic plasticity are likewise normal in *rut* and *PKA-C1* transheterozygotes. In sharp contrast, transheterozygous mutants of *rut* and *Epac*, *Rap1*, or *Vav* all lose tetanic augmentation and PTP (Fig. 9, E–H). Given the essential role of Rut-dependent cAMP signaling in mediating PTP as well as the requirements for Epac, Rap1, and Vav in FSK-induced synaptic potentiation and RP mobilization, these results support the model that Rut-cAMP-Epac signaling mediates synaptic augmentation and PTP through the Rap1-Vav pathway.

To corroborate the differential roles of Rut-dependent cAMP signaling and Abl signaling in synaptic potentiation and Gbb-induced macropinocytosis, we first tested the impacts of *rut*^*1*^ and FSK application on Gbb-induced presynaptic macropinocytosis (Fig. S5, G and H). Levels of presynaptic macropinocytosis induced by Gbb (50 ng/ml) are comparable in *rut*^*1*^ mutants and WT controls. Furthermore, prior application of FSK by itself fails to induce presynaptic macropinocytosis and has no effect on Gbb-induced macropinocytosis, supporting the fact that Rut-dependent cAMP signaling does not contribute to Gbb-induced presynaptic macropinocytosis. We next tested the ability of the Abl activator DPH to potentiate transmission. Application of the Abl activator DPH fails to mimic the action of FSK to enhance EJP amplitudes (Fig. S5, I and J). These results are consistent with differential roles for Rut-dependent cAMP signaling and Abl signaling in synaptic potentiation and Gbb-induced macropinocytosis.

### Rap1-dependent synaptic potentiation requires the F-actin cytoskeleton

**Figure 10.**
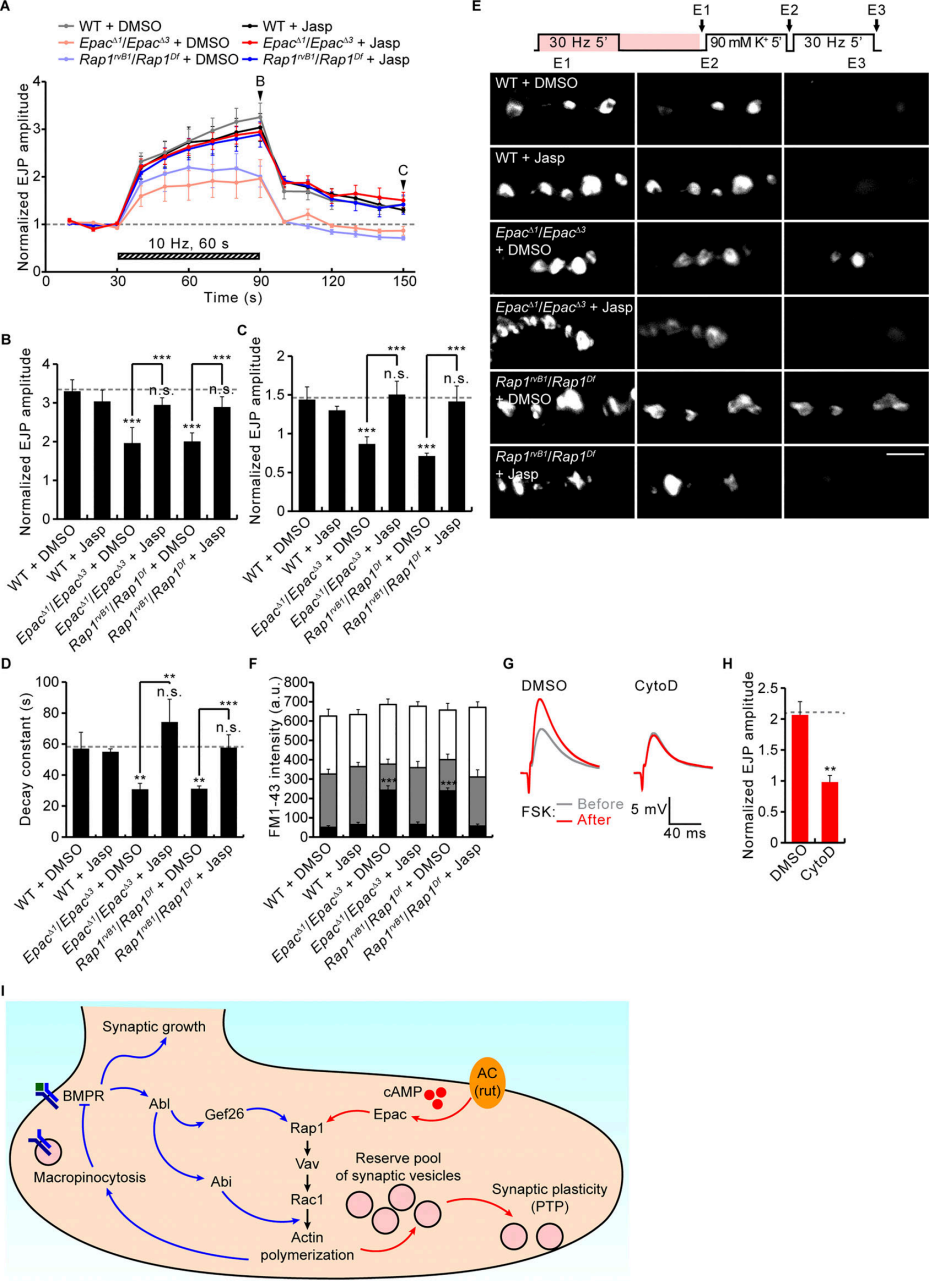
**Synaptic potentiation induced by FSK and mediated by Rap1 depends on F-actin. (A)** Normalized EJP amplitudes from WT, *Epac*^*Δ1*^/*Epac*^*Δ3*^, and *Rap1*^*rvB1*^/*Rap1*^*Df*^ third instar with/without actin polymerizing jasplakinolide (Jasp, 10 μM, 30 min; 0.1% DMSO) subjected to the PTP paradigm (Fig. 5 A). Each point indicates the mean normalized amplitude of consecutive EJPs recorded every 10 s. **(B–D)** Bar graphs of mean EJP amplitudes at the end (B) and 60 s after (C) tetanic stimulation, and PTP decay time constants (D). *n* = 12 NMJs. **(E)** FM1-43 loading–unloading protocol and representative images of NMJ boutons after ECP-RP loading (E1), high K^+^-induced ECP unloading (E2), and 30 Hz-induced RP unloading (E3; as in Fig. 5 F). **(F)** FM1-43 fluorescence intensity in boutons after ECP-RP loading (whole columns), ECP unloading (gray columns), and RP unloading (black columns). *n* = 12 boutons. **(G)** Representative EJP recordings before (gray) and after (red) FSK stimulation (10 μM, 10 min) with/without inhibition in CytoD (10 μM in 0.1% DMSO) for 40 min. **(H)** EJP potentiation by FSK in the absence/presence of CytoD. *n* = 12 NMJs. **(I)** Model for differential regulation of synaptic growth and PTP by Rap1-Vav-Rac1 signaling. Data represent mean ± SEM. Comparisons are with DMSO-treated WT, unless otherwise indicated. **, P < 0.01; ***, P < 0.001; n.s., not significant (B–D and F: one-way ANOVA with multiple comparisons; H: two-sided unpaired Student’s *t* test). Scale bar: 5 μm.

Tetanic stimulation-induced presynaptic RP vesicle mobilization and PTP require actin polymerization mediated by the Vav-Rac1-SCAR pathway (Park et al., 2022). To further test whether cAMP and Rap1 signaling act via the Vav-Rac1-SCAR pathway, we next investigated the involvement of F-actin polymerization in Epac/Rap1-mediated RP mobilization and PTP. Specifically, we tested the ability of the actin polymerization activator jasplakinolide (Jasp) to rescue synaptic defects in *Epac* and *Rap1* mutants. We found that the prior application of Jasp (10 μM) does not affect neurotransmission strength (basal EJP amplitudes) but almost completely rescued the RP vesicle mobilization and PTP plasticity defects in both *Epac* and *Rap1* mutants (Fig. 10, A–F). In the other direction, the actin polymerization inhibitor cytochalasin D (CytoD), at a low concentration (10 μM), likewise does not significantly affect basal EJP amplitudes but completely blocks the FSK-, 8-pCPT-, Rap1^CA^-, and Vav^CA^-induced potentiation of basal EJP amplitudes (Fig. 10, G and H; and Fig. S5, C and D). Taken together, these findings strongly support the model of Rut-cAMP-Epac-Rap1 signaling, mediating post-tetanic synaptic potentiation by driving Vav-Rac1-SCAR-dependent actin cytoskeleton polymerization within the presynaptic terminal.

## Discussion

At the *Drosophila* NMJ model glutamatergic synapse, presynaptic Vav-Rac1 signaling triggers actin polymerization, driving BMP-induced presynaptic macropinocytosis and activity-induced RP vesicle mobilization, two spatiotemporally distinct membrane trafficking mechanisms regulating synaptic growth and functional potentiation, respectively (Park et al., 2022). The present study provides genetic evidence that the small GTPase Rap1 operates upstream of Vav-Rac1 signaling in both of these two actin-dependent processes. Our findings indicate that Rap1 is a key regulatory node for the presynaptic actin dynamics that independently controls Gbb-induced macropinocytosis and tetanus-induced RP mobilization. We found that BMP-induced activation of Abelson (Abl) kinase is coupled to the induction of presynaptic macropinocytosis via the Gef26-Rap1 pathway during synaptic growth. We also found that Rut-dependent cAMP signaling plays a direct obligatory role in tetanus-induced RP mobilization and PTP, and that this action of cAMP signaling is mediated via the Epac-Rap1 pathway. Thus, this work demonstrates two distinct signaling mechanisms that independently control Rap1/Vav/Rac1-dependent actin cytoskeleton remodeling regulating structural and functional presynaptic plasticity.

Macropinocytosis is initiated by Rac1-SCAR/WAVE signaling membrane ruffles that subsequently develop into macropinocytic cups and intracellular macropinosomes (Buckley and King, 2017; Fujii et al., 2013). Abl kinase and Vav play central roles in this actin-dependent process. In mammalian cells, Abl phosphorylation of SCAR2/3 promotes membrane ruffling (Leng et al., 2005; Sossey-Alaoui et al., 2007; Stuart et al., 2006). At the *Drosophila* NMJ, Abl phosphorylation of SCAR complex Abi drives presynaptic macropinocytosis induced by BMP ligand Gbb (Kim et al., 2019), and the Rho GEF Vav acts upstream of Rac1 in this process (Park et al., 2022). In this study, we provided multiple lines of evidence that Vav is a major Abl target during Gbb-induced, Rac1-mediated presynaptic macropinocytosis. First, induced Abl kinase activity from a constitutively active variant or DPH application alone is sufficient to induce presynaptic macropinocytosis even in the absence of Gbb signaling. Second, full activation of Abl kinase activity almost completely occludes the action of Gbb, further supporting a role for Abl as the key mediator of Gbb-induced macropinocytosis. Third, loss of Gef26 or its downstream effector Rap1 results in impairments of both Gbb-induced and DPH-induced macropinocytosis. Finally, consistent with a critical role for presynaptic macropinocytosis in restraining BMP signaling and presynaptic growth (Kim et al., 2019), genetic interaction tests suggest that the Gef26-Rap1 pathway connects Abl kinase activation with Vav-Rac1 signaling in the restraint of presynaptic growth. Together, these results indicate that Abl activates Vav-Rac1-SCAR signaling via the Gef26-Rap1 pathway to drive Gbb-induced presynaptic macropinocytosis, thereby restraining synaptic growth. However, the molecular mechanism underlying the Abl activation of Gef26 has not yet been determined.

Despite synaptic overgrowth, the basal EJP and spontaneous mEJP amplitudes in a physiological condition (1.5 mM Ca^2+^) are not significantly altered in *Rap1* mutants compared with controls. However, the frequency of mEJPs is significantly increased in *Rap1* mutants. Interestingly, a selective increase in mEJP frequency, but not EJP and mEJP amplitudes, is also observed in *Abl* and *Vav* mutants (Lin et al., 2009; Park et al., 2022), as well as in animals depleted of presynaptic CtBP and Rabankyrin (unpublished data), two key regulators of macropinocytosis. Thus, it is highly plausible to propose that genetic conditions impairing presynaptic BMPR macropinocytosis commonly cause synaptic overgrowth, thereby leading to parallel increases of functional release sites and mEJP frequency.

In many model synapses, the presynaptic elevation of cAMP has been found to facilitate neurotransmitter release for sustained periods, leading to short- or long-term increases in neurotransmission strength (Beaumont and Zucker, 2000; Chavez-Noriega and Stevens, 1994; Cheung et al., 2006; Fernandes et al., 2015; Gekel and Neher, 2008; Salin et al., 1996). Studies of mammalian cerebral and cerebellar synapses have suggested that PKA is the major target of this cAMP action (Huang and Kandel, 1996; Salin et al., 1996; Weisskopf et al., 1994). More recent studies, however, have shown that the effects of cAMP on transmitter release are independent of PKA. For example, Epac2 has been found to partly mediate cAMP-induced potentiation at the mossy fiber-CA3 synapse of the mouse hippocampus (Fernandes et al., 2015; Gekel and Neher, 2008). In addition, I_h_ channels and Epac, but not PKA, have been found to contribute to cAMP-induced potentiation at the crayfish NMJ (Beaumont and Zucker, 2000; Zhong and Zucker, 2005), and Epac activation is shown to fully mediate cAMP-induced potentiation at the calyx of Held in the mammalian brain stem (Kaneko and Takahashi, 2004). Thus, the role of cAMP in the regulation of presynaptic transmitter release appear largely independent of PKA. Here, we provide evidence that Epac is the major target of cAMP-induced potentiation of synaptic transmission at the *Drosophila* NMJ. We show that the potentiation of EJP amplitudes by the adenylyl cyclase activator FSK is completely blocked by the loss of Epac but not by PKA and I_h_ channel activity. We also show that this modulation is fully mimicked by an Epac-specific cAMP analog.

Our findings further suggest that this FSK/cAMP-induced potentiation and PTP appear to share a common mechanism. Both potentiation processes display similar decay time constants and are associated with increases in the synaptic vesicle *P*_*r*_, readily releasable pool (RRP) size, and replenishment rate. Moreover, as previously demonstrated for PTP (Kim et al., 2009; Park et al., 2022), we find here that FSK-induced potentiation is completely blocked by the MLCK inhibitor ML-7, which interferes with the mobilization of RP SVs (Kim et al., 2009; Verstreken et al., 2005). Importantly, the prior application of FSK completely occludes the manifestation of PTP. These findings, together with results showing that genetic ablation or pharmacological inhibition of the Rut adenylate cyclase as well as Epac disrupts PTP, indicate that activation of Rut-cAMP-Epac signaling plays a direct, obligatory role in this functional potentiation at the *Drosophila* NMJ. In comparison with these findings, cAMP-PKA signaling has been implicated in a presynaptic tetanic stimulation-induced long-term potentiation (LTP) at mammalian hippocampal synapses (Frey et al., 1993; Huang and Kandel, 1996; Otmakhova et al., 2000; Salin et al., 1996; Weisskopf et al., 1994). These functional time course comparisons across species are compelling. However, the mechanisms by which cAMP signaling may play differential roles in short-term versus long-term synaptic potentiation at different synapses remain unclear.

Vav acts via the actin-regulatory Rac1-SCAR pathway to mediate tetanus-induced RP mobilization and PTP (Park et al., 2022). Consistent with previous results showing that Epac is a direct activator of Rap1 (de Rooij et al., 1998; Enserink et al., 2002; Kawasaki et al., 1998), our new findings indicate that the Rut-cAMP-Epac pathway signals through Rap1 to mediate Vav/Rac1-dependent RP mobilization and PTP. We found that the loss of Rap1 impairs tetanus-induced RP mobilization and PTP, mimicking the *rut*, *Epac*, *Vav*, and *Rac1* loss-of-function phenotypes. Moreover, dosage-sensitive transheterozygous interactions between *rut* and *Epac*, *Rap1*, or *Vav* during PTP expression, strongly indicate that Epac, Rap1, and Vav work together in a common, Rut-dependent cAMP pathway. Furthermore, we found that neuronal overexpression of constitutively active Rap1 and Vav fully mimics the effects of FSK/8-pCPT on neurotransmission, with FSK/8-pCPT-induced potentiation being almost completely abolished in *Rap1* and *Vav* mutants. These results strongly indicate that Rap1-Vav signaling acts downstream of Epac to facilitate transmitter release. Finally, as previously demonstrated for both *Vav* and *Rac1* mutants (Park et al., 2022), the RP mobilization and PTP defects in *Epac* and *Rap1* mutants are completely rescued by stabilizing filamentous actin (F-actin) with jasplakinolide (Jasp). Taken together, this work indicates that Rut-cAMP-Epac signaling acts through the actin-regulatory Rap1-Vav pathway to mediate tetanus-induced RP mobilization and PTP.

Our data indicate that Abl-Gef26 signaling does not contribute to functional presynaptic plasticity. Both tetanus-induced RP mobilization and PTP are not affected by acute pharmacological blockade of Abl kinase activity and in *Gef26* mutants. We also find that loss of Abl or Gef26 function does not impair FSK/8-pCPT-induced synaptic potentiation. Moreover, in contrast to FSK/8-pCPT, the Abl activator DPH fails to potentiate synaptic transmission. On the other hand, our data indicate that Rut-cAMP-Epac signaling plays no role in regulating structural presynaptic plasticity. We find that Gbb-induced presynaptic macropinocytosis is not impaired in *rut*^*1*^ mutants. We also find that, in contrast to DPH, FSK fails to induce presynaptic macropinocytosis in the absence of Gbb. Finally, *Epac* mutants show normal levels of Gbb-induced presynaptic macropinocytosis and do not interact with the Abl-Gef26-Rap1 pathway during synaptic growth. Thus, this work demonstrates the differential roles of the Abl/Gef26- and Rut/cAMP/Epac-mediated Rap1 pathways at the *Drosophila* NMJ (Fig. 10 I). Consistent with this conclusion, we find enrichment of Rap1 expression at the periactive zone, where endocytosis occurs, as well as in CSP-associated SV compartments. Given that mammalian Rap1 signals long-term depression via lysosomal p38MAPK (Zhang et al., 2018), it is of paramount importance to define the detailed mechanisms by which Rap1 achieves signal diversity and specificity to mediate different forms of synaptic plasticity.

How might Rut-cAMP-Epac signaling mediate PTP at the cellular level? The strong correlation between tetanus-induced RP mobilization and PTP (Kim et al., 2009; Kuromi and Kidokoro, 2003), coupled with the essential roles of Vav-Rac1 signaling and F-actin cytoskeleton networks in RP mobilization (Kuromi and Kidokoro, 2003; Park et al., 2022), implies that Epac-dependent cAMP signaling acts by promoting Rap1/Vav/Rac1-dependent actin polymerization and thus SV mobilization via actin-based molecular motors. This scenario is in general agreement with previous reports showing that RP mobilization and PTP depend on the activity of MLCK (Kim et al., 2009; Park et al., 2022; Verstreken et al., 2005), a major activator of myosin motor activity (Kamm and Stull, 2001). It remains unclear, however, whether RP mobilization per se is sufficient to increase neurotransmitter release in the absence of tetanic stimulation or whether RP mobilization simply confers competence on synapses to manifest PTP. In the latter case, Epac and Rap1/Vav/Rac1 signaling may directly act on the components of the presynaptic release machinery to increase RRP size and/or vesicular *P*_*r*_, two presynaptic parameters that ultimately determine the amount of transmitter release. In mammals, the Epac regulatory role in exocytosis is best characterized in studies of insulin secretion by pancreatic β-cells (Gloerich and Bos, 2010). Epac mediates the cAMP-induced potentiation of insulin secretion via interaction with Rab3-interacting molecule 2 (RIM2) (Kashima et al., 2001; Ozaki et al., 2000), a protein family also involved in regulating synaptic vesicle exocytosis (Wu et al., 2023). Thus, it will be interesting to determine whether, in addition to mobilizing RP vesicles, Epac/Rap1 signaling acts on the presynaptic release machinery to mediate PTP. In conclusion, this study demonstrates that Abl-Gef26 and Rut-cAMP-Epac pathways converge on actin-regulatory Rap1-Vav signaling to mediate presynaptic plasticity.

## Materials and methods

### *Drosophila* stocks

The *w*^*1118*^ strain was used as the wild-type (WT) control. *Rap1*^*rvB1*^, *UAS-Rap1,* and *UAS-Rap1*^*V12*^ (*Rap1*^*CA*^) were obtained from Jocelyn McDonald (Boettner et al., 2003; Hariharan et al., 1991; Kansas State University, Manhattan, KS, USA); *Vav*^*2*^ was obtained from Maria Martin-Bermudo (Malartre et al., 2010; University Pablo de Olavide, Sevilla, Spain); and *Gef26*^*Δ6*^ was obtained from Steven Hou (Wang et al., 2006; National Cancer Institute, Frederick, MD, USA). The *UAS-HA-abi*^*4YE*^ line has been described (Kim et al., 2019). The fly lines *GFP-Rap1* (RRID: BDSC_99942), *UAS-Bcr-Abl* (RRID: BDSC_9571), *Abl*^*1*^ (RRID: BDSC_3554), *Abl*^*4*^ (RRID: BDSC_3553), *Df(2L)BSC5* (*Gef26*^*Df*^; RRID: BDSC_6299), *Epac*^*Δ1*^ (RRID: BDSC_78799), *Epac*^*Δ3*^ (RRID: BDSC_78799), *Df(3L)ED4287* (*Rap1*^*Df*^; RRID: BDSC_8096), *Pka-C1*^*H2*^ (*PKA*^*H2*^; RRID: BDSC_4101), *Ih*^*e01599*^ (RRID: BDSC_17970), *Ih*^*f03355*^ (RRID: BDSC_85660), *UAS-Abl* (RRID: BDSC_28993), *UAS-Abl*^*K417N*^ (RRID: BDSC_8566), *rut*^*1*^ (RRID: BDSC_9404), *UAS-Pka-R1.BDK* (*UAS-PKAinh*^*1*^; RRID: BDSC_35550), *UAS-Pka-C1*^*RNAi*^ (RRID: BDSC_31599), *UAS-Pka-C2*^*RNAi*^ (RRID: BDSC_55859), *UAS-Pka-C3*^*RNAi*^ (RRID: BDSC_39050), and *UAS-Ih*^*RNAi*^ (RRID: BDSC_29574) were obtained from the Bloomington *Drosophila* Stock Center, and the *UAS-PACα* line was from the Korea *Drosophila* Resource Center (KDRC_1013). *UAS-Rap1*^*RNAi*^ (v110757) was obtained from the Vienna *Drosophila* Resource Center. GAL4 driver lines for tissue-specific expression of UAS transgenes included *C155-GAL4* (RRID: BDSC_458; (Lin and Goodman, 1994), *OK6-GAL4* (RRID: BDSC_64199; (Aberle et al., 2002), *nSyb-GAL4* (RRID: BDSC_51635; (Pauli et al., 2008), *BG57-GAL4* (Budnik et al., 1996), and *elav-GS-GAL4* (RRID: BDSC_43642; (Osterwalder et al., 2001).

Animals were maintained at 25°C on a standard cornmeal-agar medium supplemented with baker’s yeast. For experiments using the Gene Switch (GS) system, embryos carrying *elav-GS-GAL4* were collected on grape juice plates with yeast paste at the center and then raised further on a standard medium containing 10 μg/ml RU486 (Mifepristone; Sigma-Aldrich). For optogenetic control experiments, flies were crossed and the vials were wrapped in aluminum foil to minimize light stimulation during rearing. The GAL4/UAS expression system was used to drive transgene expression in specific cell types. All experiments were performed with animals at the third instar larval stage. Females were used for all experiments except those involving hemizygous *Vav* and *rut* males in Figs. 4, 8, and 9.

### Gbb-conditioned medium

*Drosophila* S2R+ cells (RRID: CVCL_Z831) were obtained from the *Drosophila* Genomics Resource Center (DGRC) and maintained at 25°C in Schneider’s medium (Sigma-Aldrich) supplemented with 10% heat-inactivated fetal bovine serum (Gibco) and a mixture of penicillin (60 μg/ml) and streptomycin (100 μg/ml) (Welgene). To generate stable Gbb-expressing cell lines, full-length *gbb* cDNA was PCR-amplified from *pAc-gbb* (Kim et al., 2019) and cloned into the vector pAc5-STABLE2-Neo (RRID: Addgene_32426) using the following primers 5′-CCC​GGT​ACC​GCC​ACC​ATG​TCG​GGA​CTG​CGA​AAC-3′ (forward, *Acc65I-gbb*) and 5′-CCC​GCG​GCC​GCT​CAA​TGG​CAC​CCG​CAG​GAT​TTC​AC-3′ (reverse, *NotI-gbb*). The PCR products were excised with Acc65I and NotI and directly inserted into the Acc65I/NotI sites of the pAc5-STABLE2-Neo vector (Addgene) to produce *pAc-gbb-GFP-NeoR*. S2R+ cells were transfected with *pAc-gbb-GFP-NeoR* using Cellfectin II (Gibco), according to the manufacturer’s instructions, and incubated in a normal medium for 72 h. The cells were treated with 600 μg/ml G418 (InvivoGen) for 72 h and with 2 mg/ml G418 for 18 days. Cells were split if necessary and the selective medium was changed every 5–6 days. At 3 wk after transfection, the cells were seeded into 96-well plates to isolate single-cell-derived colonies resistant to G418. The selection procedure was monitored by assessing GFP expression. To produce a Gbb-conditioned medium, stably transfected S2R+ cells were incubated in serum-free Schneider’s medium for 120 h. The cell debris was removed by centrifugation at 4,000 *g* for 5 min at 4°C, and the concentration of Gbb was measured as described (Kim et al., 2019).

### Immunohistochemistry imaging

Wandering third instar larvae were dissected in ice-cold Ca^2+^-free HL3 saline (70 mM NaCl, 5 mM KCl, 20 mM MgCl_2_, 10 mM NaHCO_3_, 115 mM sucrose, 5 mM trehalose, 5 mM HEPES, pH 7.2) and fixed in Bouin’s solution (Sigma-Aldrich) for 10 min. Fixed larval fillets were washed three times for 10 min each with PBS containing 0.1% Triton X-100 (PBST-0.1) and incubated overnight at 4°C in 0.2% BSA/PBST-0.1 containing primary antibodies. The following primary antibodies were used in this study: mouse anti-Brp (nc82; RRID:AB_2392664; DSHB) at 1:10, mouse anti-Dlg (4F3; RRID: AB_528203; DSHB) at 1:200, mouse anti-CSP (1G12; RRID: AB_528184; DSHB) at 1:200, FITC-conjugated goat anti-HRP (RRID: AB_2314647; Jackson ImmunoResearch Laboratories) at 1:200, Alexa Fluor 647-conjugated goat anti-HRP (RRID: AB_2338967; Jackson ImmunoResearch Laboratories) at 1:200, and Alexa Fluor 488-conjugated rabbit anti-GFP (RRID: AB_221477; Invitrogen) at 1:200. Samples were washed three times with PBST-0.1 and then incubated for 2 h at room temperature in 0.2% BSA/PBST-0.1 containing Cy3-conjugated secondary antibodies for anti-mouse (1:200; RRID: AB_2340813; Jackson ImmunoResearch Laboratories). Fluorescent images of NMJs 6/7 were acquired with a Zeiss LSM 800 laser-scanning confocal microscope using a C-Apo 40× 1.20 W (to quantitate bouton number) or Plan-Apo 63× 1.25 Oil objective at 25°C using Zen 3.4 software. To quantitate bouton number, Z-stack images of the entire NMJ 6/7 in abdominal segment 2 (A2) were collected with 1-μm spacing, and the maximum-intensity projection images were reconstructed using Zen 3.4 software (Zeiss). A satellite bouton was defined as a single bouton that was not included in a chain of boutons.

### Dextran uptake

Semi-intact preparations of wandering third instar larvae were obtained by making a dorsal incision in ice-cold Ca^2+^-free HL3 saline as described (Frank et al., 2006). Preparations were pulsed through this incision with 2 mg/ml of TMR-Dex (Molecular Probes) in a Gbb-conditioned medium (50 ng/ml final concentration) for 5 min. After complete dissection, pulsed preparations were fixed in 4% formaldehyde/PBS for 30 min and stained with FITC-conjugated goat anti-HRP as described above. Z-stack images of the entire NMJ 6/7 in the A2 segment were acquired with 1-μm spacing on the Zeiss LSM 800 microscope using a Plan Apo 63× 1.4 Oil objective at 25°C using Zen 3.4 software. The number of TMR-Dex-positive puncta (>0.2 μm in diameter) per three terminal boutons at each NMJ branch was measured on images reconstructed by maximum-intensity projection.

### FM1-43 labeling

FM1-43FX (Invitrogen) was loaded and unloaded as described (Kuromi and Kidokoro, 2000; Park et al., 2022). Briefly, wandering third-instar larvae were dissected in Ca^2+^-free HL3 saline. To load ECP and RP vesicles with FM1-43FX, synaptic boutons were electrically stimulated in HL3 saline (2 mM Ca^2+^) with 4 μM dye for 5 min at 30 Hz and further incubated in the same bath for 5 min without electrical stimulation. Excessive dye was removed by washing three times with Ca^2+^-free HL3 saline. FM1-43FX-labeled ECP vesicles were unloaded by exposing synaptic boutons for 5 min to high-K^+^ HL3 saline, with NaCl concentration reduced to maintain osmolarity (40 mM NaCl, 90 mM KCl, 20 mM MgCl_2_, 2 mM CaCl_2_, 10 mM NaHCO_3_, 115 mM sucrose, 5 mM trehalose, 5 mM HEPES, pH 7.2) (Vasin et al., 2014). FM1-43FX-labeled RP vesicles were unloaded by restimulating synaptic boutons in HL3 saline for 5 min at 30 Hz. Preparations were imaged on an upright fluorescence microscope (Axio Imager D1; Zeiss) equipped with an Axiocam 506 monochrome camera and a Plan-Apo 63× 1.0 W objective at 25°C using Zen 3.4 software. The fluorescence intensity of each preparation was assessed in three type-Ib boutons with an area >3 μm^2^. Background fluorescence intensity was subtracted using Zen 3.4 software (Zeiss).

### Synaptic electrophysiology

Third instar larvae were dissected in Ca^2+^-free, modified HL3 saline containing reduced MgCl_2_ (70 mM NaCl, 5 mM KCl, 10 mM MgCl_2_, 10 mM NaHCO_3_, 115 mM sucrose, 5 mM trehalose, 5 mM HEPES, pH 7.2). All electrophysiological recordings were made from muscle 6 in the A3 segment in modified HL3 with Ca^2+^ concentrations specified in the figure legends. The resistance of recording electrodes filled with 3 M KCl was <25 MΩ; only cells with an initial resting membrane potential below −65 mV and an input resistance above 5 MΩ were analyzed. Data were acquired and analyzed using a Neuroprobe Amplifier (Model 1600; A-M Systems), LabVIEW 14 software (National Instruments), and Clampfit 11.1 software (Molecular Devices). EJPs were elicited by applying a 500-μs pulse to the cut end of motor axons using the programmable Master-8 stimulator (AMPI). Data involving >30 EJP events were analyzed using MATLAB R2020a software (MathWorks). Miniature EJPs (mEJPs) were recorded in the absence of stimulation and analyzed with Mini Analysis 6.0.7 software (Synaptosoft). The quantal content of each NMJ was calculated as mean EJP amplitude divided by mean mEJP amplitude.

To estimate the decay time constants for FSK-induced potentiation and PTP, EJP amplitudes in the decaying phase were fitted to the first-order exponential decay equation:

$$EJP_{t}=Ae^{-t/\tau }+EJP_{0}\text{,}$$

where *EJP*_*t*_ represents the amplitude of EJP at a given time *t*, *A* represents the potentiation factor, τ is the decay time constant of potentiation, and *EJP*_*0*_ is the initial mean amplitude of EJPs before potentiation.

The sizes of the cycling (or ECP) and total synaptic vesicle pools were estimated as described with slight modifications (Kim et al., 2009; Park et al., 2022). Briefly, NMJ preparations were preincubated in HL3 saline (2 mM Ca^2+^) containing 1 μM folimycin and 100 μM dynasore for 30 min (to block recycling of transmitter-containing SVs), and motor nerves were continuously stimulated for 20 min in the same bath at 3 Hz (to estimate ECP size) or 10 Hz (to estimate total vesicle pool size). Martin’s correction for nonlinear summation was applied to all measurements of quantal content, and cumulative plots of quantal content versus time were created. To estimate the total vesicle pool size, the synaptic depression curves obtained at 10 Hz were integrated. To estimate ECP size, a cumulative plot of released quanta versus stimulation (3 Hz) time was generated, and a linear regression line fitted to points between 900 and 1,200 s was back-extrapolated to time zero. The number of ECP vesicles was estimated as the value of the y-intercept.

Readily-releasable vesicle pool (RRP) size and vesicular release probability were estimated by recording a nerve-evoked train of excitatory junctional currents (EPSCs) in a two-electrode voltage clamp (TEVC) configuration as described (Goel et al., 2019). Briefly, muscles were clamped at −70 mV with an Axoclamp 900A (Molecular Devices), and EJCs were evoked with a 60 Hz, 30 stimulus train. Nerves were stimulated with an Axon Digidata 1550B (Molecular Devices) and data were acquired and analyzed using Clampex 11.2 and Clampfit 11.1 software (Molecular Devices), respectively. Quantal content was calculated by dividing the amplitude of each EJC by the mean amplitude of mini EJCs (mEJCs). Cumulative plots of quantal content versus stimulation time were generated and a linear regression line fitted to the last 12 of 30 quantal content measurements was back-extrapolated to time zero. The y-intercept was defined as the estimated RRP size and the ratio of the amplitude of the first EJC to RRP was indicative of vesicular release probability. For estimation of RRP size before and after PTP, EJCs were evoked in 0.3 mM Ca^2+^ saline with a 60 Hz, 120-stimulus train, and a linear regression line fitted to the last 48 of 120 quantal content measurements was back-extrapolated. For estimation of RRP size by hypertonic challenge, sucrose was bath applied using a glass pipette at the anterior end of NMJs 6/7 (500 mM sucrose in Ca^2+^-free HL3 solution for 3 s). To estimate RRP size, the total sucrose charge was divided by the mean mEJC charge.

### Adenylyl cyclase optogenetics

Flies for optogenetics experiments were reared in vials wrapped in aluminum foil to minimize light stimulation during development. Third-instar larvae were dissected under red halogen light and the preparation was kept in the dark for the rest of the procedure. The preparation was exposed to a 10 Hz Blue LED pulse train at 200 μs pulse width for 1 min during a 0.5 Hz electrical nerve stimulation as described above to measure the change in EJP amplitude.

### Pharmacological reagents

Where indicated, NMJ preparations were treated with 10 μM 5-(1,3-diaryl-1H-pyrazol-4-yl)hydantoin (DPH; Sigma-Aldrich), 50 μM imatinib (Sigma-Aldrich), 1 μM folimycin (Sigma-Aldrich), 100 μM dynasore (Sigma-Aldrich), 100 μM Rp-cAMPS (Sigma-Aldrich), 20 μM ESI-09 (Tocris), 5 μM PKI-(14-22)-amide (Sigma-Aldrich), 10 μM FSK (FUJIFILM Wako Chemicals), 100 μM 8-pCPT-2′-O-Me-cAMP (8-pCPT; BIOLOG), 15 μM ML-7 (Sigma-Aldrich), 10 μM cytochalasin D (CytoD; Sigma-Aldrich), and 10 μM jasplakinolide (Jasp; Invitrogen). Stocks of all chemicals were prepared in DMSO and stored at −20°C. The final concentration of DMSO was kept below 0.1% (vol/vol) in all experiments.

### Statistical analyses

Data are presented as mean ± standard error of the mean (SEM). Results in two groups were compared by two-sided unpaired Student’s *t* tests, whereas results in multiple groups were compared by one-way ANOVA, followed by Tukey’s post hoc tests. Data distribution was assumed to be normal but was not formally tested.

### Online supplemental material

Fig. S1 shows transheterozygous interactions among *Abl*, *Gef26*, *Epac*, *Rap1*, and *Vav* during presynaptic macropinocytosis and synaptic growth. Fig. S2 shows transheterozygous interactions between *Vav* and *Abl*, *Gef26*, or *Rap1* during PTP induction. Fig. S3 shows functional synaptic properties in *Rap1* mutants. Fig. S4 shows that acute blockade of Epac, but not of PKA, impairs PTP and tetanus-induced RP mobilization. Fig. S5 shows that Epac acts through the Rap1-Vav pathway to potentiate EJP amplitudes.

## Data Availability

All data are available upon reasonable request.
